# Exploring the association between visual skills and sport-specific performance in team athletes: a systematic review and meta-analysis

**DOI:** 10.3389/fphys.2026.1797347

**Published:** 2026-03-30

**Authors:** Mulin Yang, Yuqiang Guo, Feng Yang, Kewei Zhao

**Affiliations:** 1 Physical Fitness Training Research Center, China Institute of Sport Science, Beijing, China; 2 School of Physical Education, Shanghai University of Sport, Shanghai, China; 3 Graduate School, Shandong Sport University, Jinan, China

**Keywords:** athletic performance, perceptual-cognitive skills, team sports, visual perception, visual–motor coordination

## Abstract

**Background:**

Visual skills are increasingly recognized as key contributors to performance in team sports, yet the strength and consistency of their associations with sport-specific outcomes remain uncertain. To address this gap, the present review systematically synthesized empirical evidence examining how different domains of visual skill relate to sport-specific performance among team-sport athletes.

**Methods:**

PubMed, Web of Science™, MEDLINE, and SPORTDiscus databases were systematically searched from inception to October 2025 to identify studies relating visual skills to sport-specific performance in team-sport athletes. Risk of bias was conducted using a modified and validated tool for observational and correlational studies, and certainty of evidence was evaluated using the GRADE approach.

**Results:**

Of 4,118 records identified, 22 studies (*n* = 1,113, male = 954, female = 159) were included, encompassing basketball, soccer, baseball, volleyball, handball, and other team sports. The relationship between multiple object tracking and sport-specific performance was large (*r* = 0.54; 95% CI: 0.30–0.71; *p* = 0.00), while visual attention (*r* = 0.39; 95% CI: 0.16–0.53; *p* = 0.00), and visual search (*r* = 0.36; 95% CI: 0.16–0.48; *p* = 0.00), demonstrated moderate associations. Simple reaction time (*r* = −0.31; 95% CI: −0.42 to −0.19; *p* = 0.00) and choice reaction time (*r* = −0.37; 95% CI: −0.58 to −0.11; *p* = 0.01) showed moderate negative correlations with performance, indicating faster reaction speeds were associated with better performance. Visual working memory, eye-hand coordination, and inhibitory control exhibited small effect sizes. In contrast, depth perception, the only visual-perceptual skill analyzed, showed trivial and non-significant associations (*r* = 0.09; 95% CI: −0.20–0.36; *p* = 0.56).

**Conclusion:**

Visual-cognitive skills appear to show stronger links with team-sport performance, whereas basic perceptual skills contribute little. Evidence quality remains limited, and more robust, ecologically valid studies are needed to clarify causality and guide training applications.

**Systematic Review Registration:**

https://www.crd.york.ac.uk/PROSPERO/view/CRD420251171665, identifier CRD420251171665.

## 1 Introduction

Team sports are characterized by rapidly evolving and highly competitive environments in which athletes must efficiently extract, integrate, and utilize visual information. Within very short time windows, players are required to locate teammates and opponents, identify relevant cues, anticipate action intentions, and make effective technical and tactical decisions (Silva et al., 2022b; Zwierko et al., 2022; Fortin-Guichard et al., 2025). Accordingly, visual skills are considered a central determinant of sport-specific performance in team sports. Beyond their contribution to the stability and accuracy of technical execution (Lochhead et al., 2024a), visual skills also exert a direct influence on tactical decision-making quality and overall competitive performance (Memmert et al., 2009; Natsuhara et al., 2020). Sports visual skills comprise a multidimensional set of abilities, including perception, attentional, visual tracking, and cognitive operation (Erickson, 2021), which collectively determine the efficiency of visual information processing during competition (Ebrahimi et al., 2025). A substantial body of evidence (Silva et al., 2022a; Rodrigues et al., 2024a; Hernández-Peña et al., 2025; Piechota et al., 2025) indicates that expert athletes outperform novices on these visual skills. Compared with less skilled performers, experts more effectively exploit peripheral vision to detect dynamic cues and use more efficient and optimized visual search strategies to filter irrelevant information. These refined capacities for visual attentional allocation and information filtering form a critical cognitive foundation underpinning rapid and accurate decision-making in competitive contexts.

As technical complexity, game tempo, and physical intensity continue to increase in team sports, competitive environments impose progressively greater demands on athletes’ speed of information extraction and tactical judgment (Scharfen and Memmert, 2021). Therefore, identifying visual advantages in elite athletes alone is insufficient to fully capture the functional significance of visual skills in athletic performance. Further clarification of the associations between visual skills and sport-specific performance is therefore required. An increasing number of studies have examined the associations between visual skills and sport-specific performance in team sports. For instance, Gou and Li (2023) demonstrated that multiple object tracking (MOT), a key indicator of dynamic attentional allocation and tracking efficiency, was strongly associated with basketball players’ passing decisions and shot selection. Players exhibiting superior MOT performance also showed significantly higher decision-making accuracy under real competitive conditions. In volleyball, Trecroci et al. (2021) found that performance in visual search (VS) and visual attention (VA) was stably positively correlated with passing accuracy, serving quality, and change-of-direction speed. Similarly, in soccer, selective attention and inhibitory control (IC) have been shown to predict the quality of technical actions during dribbling, ball control, and physical contests, with high-level players demonstrating superior performance on these visual-cognitive tasks (Schumacher et al., 2024). In baseball, converging evidence (Laby et al., 2018; Chen et al., 2021; Morishita et al., 2025) indicates that greater accuracy in visual judgment and more efficient visual information processing are associated with superior performance in metrics such as on-base percentage, walk rate, and hitting quality. These findings suggest that visual skills are not ancillary capacities but constitute a core component of sport-specific performance in team sports. Distinct dimensions of visual skill may shape individual differences in athletic performance through their influence on technical execution quality, perceptual accuracy, and tactical decision-making efficiency.

Although the importance of visual skills for performance in team sports has been widely examined, the findings across existing studies remain inconsistent. On the one hand, visual skills encompass multiple relatively distinct subcomponents. Considerable heterogeneity in measurement systems and task paradigms across studies has resulted in marked variability in the reported strength of associations between individual visual subcomponents and sport-specific performance. On the other hand, even when examining the same visual component, findings vary across sport disciplines, competitive levels, and task contexts, thereby limiting a coherent understanding of the mechanisms through which visual skills contribute to performance. Although existing reviews have addressed aspects of this topic (Brams et al., 2019; Laby and Appelbaum, 2021; Lochhead et al., 2024b; Lochhead et al., 2024a), they have not systematically compared the magnitude of associations across distinct visual subskills. To date, no meta-analysis has comprehensively synthesized quantitative evidence on the relationships between multiple visual skills and sport-specific performance in team-sport athletes. Given the potential applied relevance of visual skills for talent identification, the development of visual training interventions, and performance prediction in team sports, a more systematic synthesis of the available evidence is warranted.

In light of these issues, the purpose of this systematic review and meta-analysis was to synthesize existing evidence on the associations between visual skills and sport-specific performance in team sports, and to quantitatively estimate and compare the magnitude of effects across different visual skill domains. In addition, the findings aim to provide an evidence-based foundation for coaches and researchers to more strategically apply visual assessment tools in talent identification, training monitoring, and performance diagnostics.

## 2 Methods

The present review was rigorously conducted following the PRISMA 2020 guidelines (Page et al., 2021) to ensure transparency, reproducibility, and methodological rigor. In addition, all procedures adhered to the established framework for systematic reviews in sport and exercise science (Gunnell et al., 2022). The review protocol was prospectively registered with the International Prospective Register of Systematic Reviews (CRD420251171665) on 19 October 2025.

### 2.1 Search strategy

**FIGURE 1:**
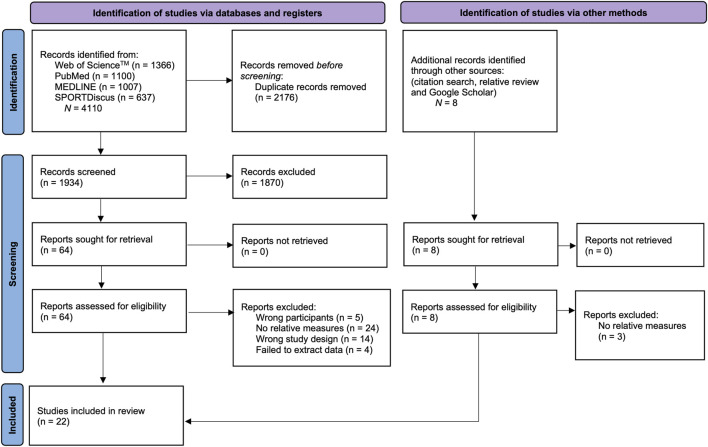
PRISMA flow chart of search process.

A comprehensive literature search was designed to identify studies examining the association between visual skills and sport-specific performance in team-sport athletes. Searches were conducted in PubMed, Web of Science Science™, MEDLINE, and SPORTDiscus from database inception to 20 October 2025. Free-text terms were using Boolean logic, truncation, and proximity operators to balance sensitivity and precision. Full database-specific strings are provided in Supplementary Appendix S2. To enhance coverage, we manually screened the reference lists of all included articles and related reviews and performed forward citation checks. Eligibility was limited to English-language, peer-reviewed publications reporting quantitative data on team-sport participants. Records were deduplicated (EndNote 20.5, Clarivate Analytics, Philadelphia, PA, USA), after which two reviewers (M.Y. and Y.G.) independently screened titles/abstracts against prespecified criteria. Disagreements were resolved by discussion with a third reviewer (K.Z.). Full texts were obtained for studies deemed potentially relevant; when necessary, corresponding authors were contacted to secure complete manuscripts. The study-selection process is summarized in Figure 1.

### 2.2 Inclusion and exclusion of studies

Eligible studies were required to provide original quantitative data, employing cross-sectional, or observational research designs without restriction on publication year. Studies published in languages other than English, as well as qualitative studies, reviews, conference abstracts, and opinion papers, were excluded.

1. Population: Studies were included if they examined team-sport athletes of any age, sex, or competitive level who were actively engaged in organized team sports such as soccer, basketball, volleyball, handball, hockey, rugby, baseball, or other comparable team games. The definition of athlete followed the criteria proposed by McKay et al. (2022), requiring participants to be competing at Tier three or higher according to their performance level classification. Studies involving non-athlete populations, individual-sport athletes, patients, or non-sporting participants (e.g., coaches or officials) were excluded.
2. Interest: According to the “Two-visual-system Hypothesis” (David et al., 2021), visual processing of athletes can be divided into two functional domains: visual-perceptual skills, mediated primarily by the ventral “vision-for-perception” pathway, and visual-cognitive skills, governed by the dorsal “vision-for-action” pathway. Visual-perceptual skills involve low-level sensory processes such as visual acuity, contrast sensitivity, gaze performance, peripheral vision and depth perception (DP), whereas visual-cognitive skills encompass high-level perceptual-cognitive and visuomotor functions, including VA, MOT, eye-hand coordination (EHC), and decision-making (Erickson, 2021). Together, these skills enable athletes to perceive, interpret, and respond effectively to complex sport-specific environments.

Sport-specific performance was defined as the execution quality or effectiveness of sport-specific tasks within team-sport contexts. Eligible studies were required to assess performance through objective or standardized measures, including but not limited to technical execution (e.g., passing accuracy), tactical decision-making (e.g., situational judgment, anticipation accuracy), or composite performance indices derived from training or competition statistics. Only on-field assessments conducted during training or competition were considered, provided that they objectively reflected athletes’ skill execution and competitive effectiveness in team sports.

3. Context: Studies conducted within the context of team sports, where athletes engaged in interactive and cooperative sporting activities that required visual information processing and collective performance.
4. Outcomes: Studies examining the association between visual skills and sport-specific performance among team-sport athletes were considered eligible.

### 2.3 Data collection process

Two reviewers (M.Y. and Y.G.) independently extracted all relevant data using a customized Excel extraction sheet (Microsoft Corporation, Redmond, WA, USA). The extraction form was pilot tested on five randomly selected studies to ensure clarity and consistency, and it was further refined through collaborative discussion among the review team before formal use. Any discrepancies identified during the extraction process were resolved through consensus discussion, and when agreement could not be achieved, a third reviewer (K.Z.) acted as an adjudicator to reach a final decision.

From each full-text article, the following information was recorded: (i) general study identification details (author, and year); (ii) study design and methodological characteristics; (iii) sample size and participant characteristics (sex, age, competitive level, and sport type); (iv) specific visual skill domains assessed (e.g., visual-perceptual skills such as visual acuity, depth perception, contrast sensitivity, or visual-cognitive skills such as visual attention, decision-making, and multiple object tracking); (v) assessment tools and measurement procedures; (vi) sport performance outcomes (e.g., technical execution, on-field decision-making skills or match statistics); (vii) details of data analysis and statistical approaches (e.g., Pearson’s or Spearman’s correlational coefficients); and (viii) reported effect sizes, confidence intervals, and exact p-values for associations between visual variables and performance outcomes.

When key data were missing or incompletely reported, corresponding authors were contacted *via* email. If no response was received, alternative contact methods (e.g., ResearchGate) were used. ResearchGate or institutional directory) were used. When numerical data were presented only in figures or graphs, quantitative values were extracted using WebPlotDigitizer website (https://automeris.io/WebPlotDigitizer) whenever possible (Burda et al., 2017). All extracted data were subsequently organized in a structured spreadsheet for verification prior to quantitative synthesis.

### 2.4 Risk of bias assessment

The Risk of bias (RoB) for each included study was assessed using a customized evaluation framework adapted from the method applied by previous systematic reviews on correlational and observational designs (Hoy et al., 2012; Van Hooren et al., 2024). The selection of items was informed by established risk-of-bias frameworks for observational research and tailored to reflect methodological characteristics specific to cross-sectional and correlational study designs in sport science. This tool evaluates key domains of methodological quality and potential sources of bias, including the clarity and justification of inclusion and exclusion criteria, adequacy of participant descriptions, representativeness and directness of data collection, validity and reliability of measurement instruments, consistency of testing conditions, control of potential confounding factors, appropriateness of statistical analyses, and an overall judgment of study level bias. Two reviewers (M.Y. and Y.G.) independently performed the RoB assessment for all included studies, and any discrepancies were resolved through discussion or adjudication by a third reviewer (K.Z.).

The certainty of evidence for each outcome was evaluated using the Grading of Recommendations, Assessment, Development and Evaluation (GRADE) approach (Atkins et al., 2004). Each outcome was initially rated as “high” and downgraded by one or more levels to “moderate,” “low,” or “very low” according to predefined criteria. Specifically, the quality was downgraded for: (1) imprecision, when the total sample size was fewer than 100 participants; (2) inconsistency, when substantial statistical heterogeneity was observed (*I* ^2^ > 50%); and (3) risk of bias, when more than 50% of the studies contributing to a pooled estimate were rated as having one or more high-risk items. For outcomes reported by only a single study, inconsistency was automatically downgraded. The GRADE assessment ensured that the interpretation of results reflected both the methodological quality and the robustness of the available evidence.

### 2.5 Statistical analysis

#### 2.5.1 Correlation extraction and calculation

All available raw data, correlation coefficients, and associated *p* values were systematically retrieved and organized within a Microsoft Excel worksheet (Microsoft Corp., Redmond, WA, USA) for subsequent analysis. When a study reported multiple indicators that could represent either visual skills or sport-specific performance, a single measure was selected to avoid redundancy. Priority was given to a composite or overall performance index (e.g., total scores of match performance) when available; otherwise, the indicator that best represented the athlete’s sport-specific performance and demonstrated the strongest relevant association was selected. Prior study (Oranchuk et al., 2024) have demonstrated that calculating Pearson’s *r* from aggregated summary statistics (e.g., means, standard deviations, or group-level data) frequently produces spuriously high correlation estimates, which can substantially bias pooled effect sizes in correlation meta-analysis. To avoid this risk, this review included only studies that explicitly reported Pearson’s or Spearman’s *r* and corresponding sample sizes, or provided access to raw data enabling the computation of exact Pearson correlations using SPSS (Version 25.0; IBM Corp., Armonk, NY, USA). Studies for which correlation coefficients could not be directly obtained or reliably computed were excluded from quantitative synthesis.

#### 2.5.2 Meta-analytical synthesis

The meta-analysis and data visualization were conducted using the “meta”, “metafor” and “ggplot2” packages in R software (version 4.3.3, R Core Team, Vienna, Austria). The meta-analysis was performed on the outcomes analysed in at least three studies. Correlation coefficients representing the association between visual skills and sport-specific performance served as the primary effect size. When studies reported Spearman’s *r*, these values were converted to Pearson’s *r* using Equation 1 (*r* = Pearson’s *r* correlation coefficient, *r*_s_ = Spearman’s *r* correlation coefficient), given that Spearman coefficients are generally smaller in magnitude than their Pearson counterparts (Rupinski and Dunlap, 1996). In all other cases, the originally reported Pearson correlations were retained for analysis.

$$r=2\sin\left(r_{s}\frac{\pi }{6}\right)$$ (1)

All reported or derived correlation coefficients were converted to Fisher’s *z* values prior to quantitative synthesis, following established meta-analytic procedures (Borenstein et al., 2009). This transformation stabilizes variance and ensures that confidence intervals are appropriately estimated within the analytic model. Fisher’s *z* was calculated using the following Equation 2 (*Z* = Fisher’s *z*, *LN* = natural log, *r* = Pearson’s *r* correlation coefficient).

$$Z=\mathrm{LN}\left(1+r\right)/\left(1‐r\right)/2$$ (2)

A random-effects model was employed to aggregate Fisher’s z values across studies. For any outcome variable assessed by two or more independent studies, a dedicated random-effects meta-analysis was performed. After pooling, Fisher’s *z* estimates were back-transformed to Pearson’s r to facilitate interpretation. Effect magnitudes were categorized as follows: <0.10 trivial, 0.10–0.29 small, 0.30–0.49 moderate, 0.50–0.69 large, 0.70–0.89 very large, and 0.90–0.99 nearly perfect (Hopkins et al., 2009).

#### 2.5.3 Assessment of heterogeneity

Heterogeneity across studies was quantified using the *I* ^*2*^ statistic, which reflects the proportion of total variability in effect estimates attributable to true between-study differences rather than sampling error. Values of *I* ^*2*^ were interpreted according to conventional thresholds, with <25% indicating low heterogeneity, 25%–50% representing moderate heterogeneity, and values exceeding 50% reflecting high heterogeneity (Higgins et al., 2003).

#### 2.5.4 Publication bias and sensitivity analyses

Potential publication bias was assessed through visual inspection of funnel plots for each meta-analysis (Sterne and Egger, 2001). Funnel plots were generated regardless of the number of included studies; however, when fewer than ten effect sizes were available, the statistical power of these plots to detect publication bias was considered insufficient, and any interpretation of asymmetry should therefore be made with caution. When at least ten effect sizes were present, asymmetry, such as uneven dispersion of effect sizes or the absence of smaller studies on one side of the plot, was considered suggestive of potential publication bias or small-study effects.

To further evaluate the stability of the pooled estimates, a leave-one-out sensitivity analysis was conducted, in which each study was sequentially removed to determine whether the overall effect was unduly influenced by any single dataset. Consistent results across iterations were interpreted as evidence of robustness in the synthesized findings.

## 3 Results

### 3.1 Charactertics of included studies

The database search across Web of Science™ (*n* = 1,366), PubMed (*n* = 1,110), MEDLINE (*n* = 1,007), and SPORTDiscus (*n* = 637) initially yielded 4,110 records, with an additional 8 records identified through other sources. After removal of 2,176 duplicates, 1934 unique records remained for screening. During title and abstract screening, 1870 studies were excluded for not meeting the eligibility criteria, leaving 64 articles for full-text assessment. Following full-text review, 47 studies were excluded due to reasons such as wrong participants (*n* = 5), no relative measures (*n* = 24), wrong study design (*n* = 14) and failed to extract data (*n* = 4). A total of 22 studies met the inclusion criteria and were included in the final qualitative synthesis, of which 22 provided sufficient data for quantitative meta-analysis. A detailed flow of study selection is presented in Figure 1.

The characteristics of the included studies are summarized in Table 1. A total of 22 studies published between 1981 and 2025 were included, encompassing 1,113 team athletes (954 male; 159 female) across basketball (Isaacs, 1981; Mangine et al., 2014; Pehar et al., 2018; Jin et al., 2020; Laby, 2020; Gou and Li, 2023; Zemková et al., 2025b), soccer (Zisi et al., 2003; van Maarseveen et al., 2018; Scharfen and Memmert, 2019; Scharfen and Memmert, 2021; Phillips and Andre, 2023; Schumacher et al., 2024; Broodryk et al., 2025), baseball (Laby et al., 2018; Chen et al., 2021; Carrol et al., 2023; Morishita et al., 2025), softball (Carrol et al., 2023), volleyball (Trecroci et al., 2021), handball (Krawczyk et al., 2019; Krawczyk and BodasiŃSki, 2020), and polo (Standing and Best, 2019) related performance contexts. The competitive level of participants ranged from sub-elite youth athletes to elite professional performers, with most samples drawn from competitive or high-performance settings. Sample sizes varied substantially, from small elite cohorts of 10–16 athletes to large-scale datasets involving over 400 players. Participant ages ranged from 11 years in youth developmental squads to over 35 years in adult professional groups, with both male-only and mixed-gender samples represented.

**TABLE 1:** Characteristics of includedstudies.

Study	Demographics	Visual skill type		Sport-specific performance
		Perceptual skills	Cognitive skills	
Isaacs (1981)	*N*= 12 (F) Basketball Sub-elite	DP: *r*= 0.54, *p*= 0.003		Free-throw shooting percentage
Zisi et al. (2003)	*N*= 45 (M) Age = 11.8 ± 0.9 years Soccer Sub-elite	DP_(1):_ *r*= 0.07, *p*> 0.05 DP_(2)_: *r*= −0.10, *p*> 0.05	SRT: *r*= −0.27, *p*> 0.05 CRT: *r*= −0.54, *p*< 0.05 VA: *r*= −0.41, *p*> 0.05	Instep kicking performance
Mangine et al. (2014)	*N*= 12 (M) Age = 19.4–30.7 years Basketball Elite		MOT: *r*= 0.78, *p*= 0.003 CRT: *r*= −0.16, *p*> 0.05	Game performance statistics (assist-to-turnover ratio)
Laby et al. (2018)	*N*= 450 (M) Baseball Elite		EHC: *r*= 0.25, *p*< 0.05	Plate discipline metrics (in-zone swing)
Pehar et al. (2018)	*N*= 88 (M) Age = 21.1 ± 3.5 years Basketball Elite		SRT: *r*= 0.28, *p*< 0.05	Basketball field agility tests
van Maarseveen et al. (2018)	*N*= 22 (F) Age = 16.3 ± 1.1 yrs Soccer Sub-elite		Anticipation: *r*= 0.138, *p*> 0.05 DM: *r*= −0.204, *p*> 0.05 VWM: *r*= 0.262, *p*> 0.05	*In-situ*small-sided game performance score
Krawczyk et al. (2019)	*N*= 12 (M) Handball goalkeeper Elite		SRT: *r*= 0.62, *p*< 0.05 CRT: *r*= 0.74, *p*< 0.05 Anticipation: *r*= 0.64, *p*< 0.05	Motor characteristics of saves
Scharfen and Memmert (2019)	*N*= 15 (M) Age = 12.7 ± 0.5 years Soccer Elite		VA_(1)_: *r*= 0.395, *p*= 0.145 VA_(2)_: *r*= 0.396, *p*= 0.143 VA_(3)_: *r*= 0.021, *p*= 0.940 VWM: *r*= 0.553, *p*= 0.033 MOT: *r*= 0.175, *p*= 0.533	Motor performance test
Standing and Best (2019)	*N*= 19 (12M/7F) Age = 36.2 ± 14.1 yrs Polo Elite		CRT: *r*= 0.020, *p*= 0.889	Polo playing handicap
Jin et al. (2020)	*N*= 24 (M) Age = 20.5 ± 2.2 years Basketball Sub-elite		MOT: *r*= 0.621, *p*< 0.01	Match-related performance (Assists)
Krawczyk and BodasiŃSki (2020)	*N*= 10 (M) Age = 30.5 ± 5.5 years Handball goalkeeper Elite		SRT: *r*= 0.621, *p*< 0.01 CRT: *r*= 0.621, *p*< 0.01 Anticipation: *r*= 0.621, *p*< 0.01	Match-related performance (successful save)
Laby (2020)	*N*= 16 (M) Basketball Elite		VA: *r*= 0.539, *p*= 0.047	Free-throw shots performance
Chen et al. (2021)	*N*= 22 (F) Age = 20–35 years Baseball Elite		VA: *r*= 0.036, *p*= 0.044 EHC: *r*= 0.098, *p*= 0.33	Batting hit accuracy
Scharfen and Memmert (2021)	*N*= 156 (M) Age = 12–34 years Soccer Elite		VWM: *r*= 0.29, *p*< 0.05 VA: *r*= 0.34, *p*< 0.05 MOT: *r*= 0.22, *p*> 0.05	Game time
Trecroci et al. (2021)	*N*= 43 (F) Age = 11.2 ± 0.8 years Volleyball Sub-elite		SRT: *r*= −0.354, *p*= 0.02 VS: *r*= −0.343, *p*= 0.025 IC: *r*= −0.169, *p*= 0.279	Volleyball-specific skills
Carrol et al. (2023)	*N*= 26 (10M/16F) Age_(M)_= 20.3 ± 1.3 years Age_(F)_= 20.6 ± 1.0 years Baseball and softball Sub-elite		IC: *r*= −0.174, *p*> 0.05 VS: *r*= −0.156, *p*> 0.05	Batting average
Gou and Li (2023)	*N*= 24 (F) Age = 20.3 ± 2.3 years Basketball Sub-elite		MOT: *r*= 0.711, *p*< 0.01	Passing performance
Phillips and Andre (2023)	*N*= 13 (F) Age = 18–22 years Soccer Elite		MOT: *r*= −0.380, *p*= 0.200 VA: *r*= 0.650, *p*= 0.016	Passing accuracy
Schumacher et al. (2024)	*N*= 30 (M) Age = 13.3 ± 0.8 years Soccer Sub-elite		SRT: *r*= −0.408, *p*< 0.05 VA: *r*= 0.572, *p*< 0.01 VS: *r*= 0.533, *p*< 0.01 IC: *r*= −0.380, *p*< 0.05 VWM: *r*= −0.125, *p*> 0.05	Incomplete pass rate
Broodryk et al. (2025)	*N*= 44 (M) Age = 24.4 ± 2.5 years Soccer Sub-elite		EHC: *r*= 0.019, *p*> 0.05	Reactive agility test
Morishita et al. (2025)	*N*= 14 (M) Age = 20.6 ± 1.0 years Baseball Elite		DM: *r*= 0.82, *p*< 0.05 VA: *r*= −0.49, *p*> 0.05	Walk percentage
Zemková et al. (2025b)	*N*= 16 (M) Age = 15.5 ± 0.9 years Basketball Sub-elite		SRT: *r*= 0.010, *p*= 0.972 CRT: *r*= 0.156, *p*= 0.579 VA: *r*= −0.116, *p*= 0.680	Game efficiency

^(1)^ *N*, number of participants; F, female; M, male.

^(2)^CRT, choice reaction time; DP, depth perception; DM, decision making; EHC, Eye-hand Coordination; IC, inhibitory control; MOT, multiple object tracking; SRT, simple reaction time; VA, visual attention; VS, visual search; VWM, visual working memory.

Across the included studies, the assessment of visual skills encompassed both perceptual visual skills (e.g., DP) and cognitive-perceptual skills (e.g., Anticipation, IC, VA and MOT). Among these, MOT, VA, simple and choice reaction time (SRT/CRT), and visual working memory (VWM) were the most frequently evaluated domains. Some studies focused exclusively on a single visual skill (e.g., EHC in baseball or anticipation in handball), whereas others incorporated a comprehensive visual-cognitive testing battery. Regarding sport-specific performance, the studies employed diverse objective measures, ranging from technical execution (e.g., shooting percentage in basketball, kicking accuracy in soccer, batting metrics in baseball) and tactical decision-making to composite performance indices derived from actual match statistics (e.g., assists, turnovers, or goalkeeper saves).

### 3.2 Risk of bias

**FIGURE 2:**
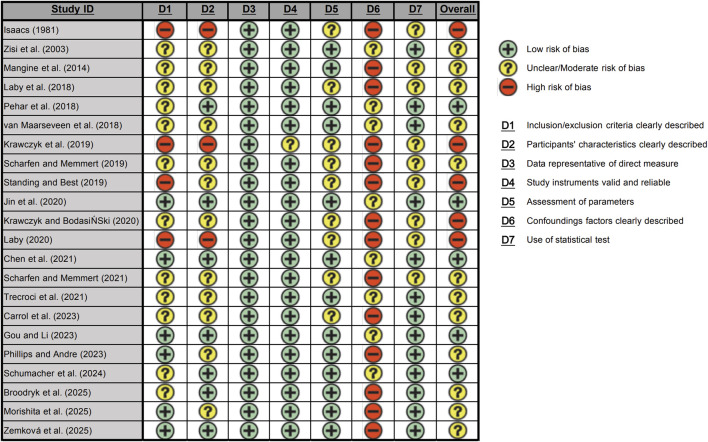
Risk of bias assessment for each study.

**FIGURE 3:**
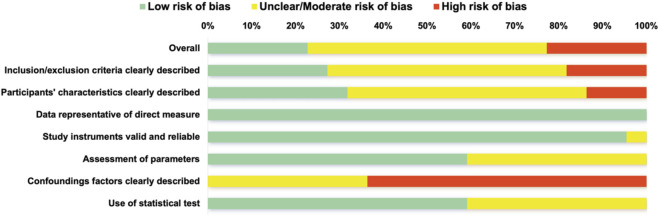
Risk of bias indicated by percentages of assessed biases across all included studies.

The risk of RoB assessment for the 22 included studies is summarized in Figures 2, 3. Overall, the methodological quality of the included literature was heterogeneous. Approximately 23% (*n* = 5) of the studies were classified as having a low overall risk of bias, while the majority (*n* = 12) exhibited a moderate risk, and the remaining 22% (*n* = 5) were deemed to have a high risk of bias. The domains related to the directness of measures (“Data representative of direct measure”) and the validity of instruments (“Study instruments valid and reliable”) demonstrated the lowest risk of bias, indicating that most studies employed robust tools to assess visual skills and sport performance. However, the control of confounding factors (“Confounding factors clearly described/controlled”) was identified as the primary source of high risk across the body of evidence. Most studies failed to measure, report, or statistically control for key covariates such as training background, positional role, visual correction status, physical maturity, and fatigue, resulting in a consistently high risk of bias for D6. Additionally, the clarity of inclusion/exclusion criteria and participant characteristics showed a mixed risk profile, with some studies lacking sufficient detail to ensure full reproducibility.

### 3.3 Meta-analysis

**FIGURE 4:**
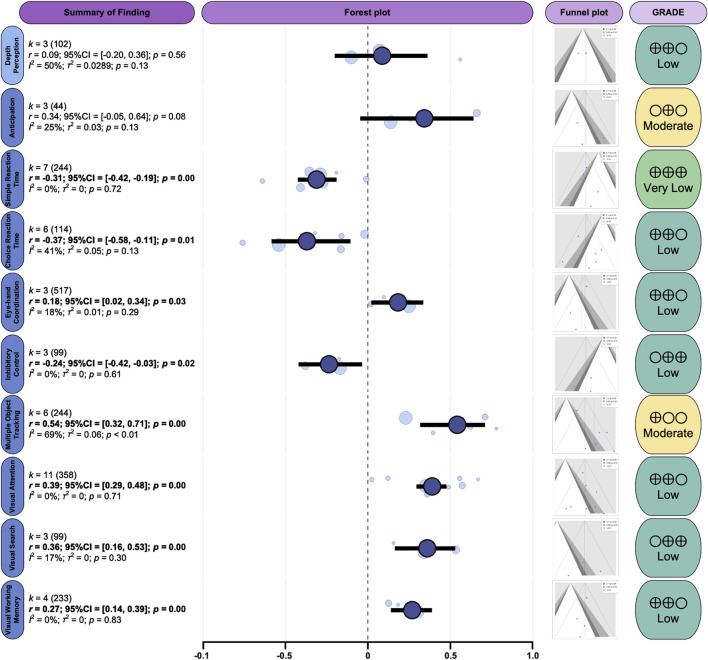
Primary pooled effect sizes for the outcomes. *k*, number of included effects; *r*, Pearson’s correlation coefficient; 95% CI, 95% confidence interval; *p*, p value; *I* ^2^, I-squared statistic, quantifying the proportion of total variation across studies that is due to heterogeneity; *τ* ^2^, Tau-squared, representing the estimated between-study variance in a random-effects meta-analysis; GRADE, Grading of Recommendations Assessment, Development, and Evaluation*,* a framework used to assess the certainty (quality) of evidence across studies.

Meta-analysis was conducted for outcomes analyzed in at least three independent studies, focusing on the most frequently assessed visual skills. The results for the primary pooled effect sizes are presented in Figure 4.

A meta-analysis of three effect sizes derived from two studies (*n* = 102) revealed a trivial and non-significant association between DP and sport-specific performance (*r* = 0.09; 95% CI: −0.20–0.36; *p* = 0.56). Between-study heterogeneity was moderate (*I* ^2^ = 50%; *p* = 0.13). According to the GRADE framework, the certainty of evidence was rated as moderate.

A meta-analysis of three effect sizes derived from three studies (*n* = 44) identified a moderate association between anticipation skill and sport-specific performance (*r* = 0.34; 95% CI: −0.05–0.64; *p* = 0.08). Moderate heterogeneity was observed among the included studies (*I* ^2^ = 25%; *p* = 0.13), indicating meaningful variation in effect estimates. According to the GRADE framework, the certainty of the evidence was rated as low.

Reaction time was examined by separating simple and choice components. The SRT meta-analysis, based on seven effect sizes from seven studies (*n* = 244), identified a moderate negative association between faster SRT and better sport-specific performance (*r* = −0.31; 95% CI: −0.42 to −0.19; *p* = 0.00). Low heterogeneity was present (*I* ^2^ = 0%; *p* = 0.72). In contrast, the CRT meta-analysis, comprising six effect sizes derived from six studies (*n* = 114), revealed a moderate negative association with performance (*r* = −0.37; 95% CI: −0.58 to −0.11; *p* = 0.01), with heterogeneity at a moderate level (*I* ^2^ = 41%; *p* = 0.13). According to the GRADE assessment, the certainty of evidence for SRT was rated as high, whereas the evidence for CRT was rated as moderate, largely due to the risk of bias.

Three independent effect sizes from three studies (*n* = 517) were synthesized to examine the association between EHC and sport-specific performance. The pooled analysis indicated a small positive correlation (r = 0.18; 95% CI: 0.02–0.34; *p* = 0.03). Heterogeneity was minimal (*I* ^2^ = 18%; *p* = 0.29), suggesting consistent findings across studies. According to the GRADE framework, the certainty of evidence was rated as moderate due to a high risk of bias in most studies.

The meta-analysis of IC, based on three effect sizes from three studies (*n* = 99), identified a small negative association with sport-specific performance (r = −0.24; 95% CI: −0.42 to −0.03; *p* = 0.03). Heterogeneity was low (*I* ^2^ = 0%; *p* = 0.61). According to the GRADE framework, the certainty of evidence was rated as moderate, primarily due to imprecision associated with the limited total sample size.

MOT was one of the most frequently investigated domains, with six effect sizes from six studies (*n* = 244) included in the meta-analysis. The pooled estimate indicated a large positive association between MOT and sport-specific performance (*r* = 0.54; 95% CI: 0.30–0.51; *p* = 0.00). Substantial heterogeneity was observed (*I* ^2^ = 69%; *p* < 0.01), indicating considerable variability across studies. The certainty of evidence was rated as low according to the GRADE framework.

The VA meta-analysis incorporated eleven effect sizes from nine studies (*n* = 358). The pooled results demonstrated a moderate positive association between VA and sport-specific performance (*r* = 0.39; 95% CI: 0.16–0.53; *p* = 0.00). Heterogeneity was low (*I* ^2^ = 0%; *p* = 0.71), indicating consistent findings across studies. Nevertheless, the certainty of evidence was rated as moderate under the GRADE framework due to the persistently high risk of bias arising from uncontrolled confounding.

The VS meta-analysis integrated three effect sizes from three studies (*n* = 99), revealing a moderate positive association with sport-specific performance (*r* = 0.36; 95% CI: 0.16–0.48; *p* = 0.00). Low heterogeneity was present (*I* ^2^ = 17%; *p* = 0.30). According to the GRADE framework, the certainty of evidence was rated as moderate.

Four effect sizes from four studies (*n* = 233) were included in the VWM meta-analysis. The pooled results indicated a small positive association between VWM skill and sport-specific performance (*r* = 0.27; 95% CI: 0.14–0.39; *p* = 0.00). Heterogeneity was low (*I* ^2^ = 0%; p = 0.15). According to the GRADE framework, the certainty of evidence was rated as also moderate.

## 4 Discussion

### 4.1 Summary of the outcomes

This study aimed to investigate the associations between visual skills and sport-specific performance in team sports. Based on data from 22 studies including 1,113 team-sport athletes, MOT demonstrated the strongest positive association with sport-specific performance (*r* = 0.54), corresponding to a large effect size. VA also showed a moderate positive association with performance (*r* = 0.39), suggesting that attentional allocation and focusing capabilities are relevant for successful performance in team sports. In addition, VS (*r* = 0.36) and VWM (*r* = 0.27) were also positively associated with sport performance, although the effect sizes were relatively smaller. Reaction time measures showed negative associations with sport performance, with both SRT (*r* = −0.31) and CRT (*r* = −0.37) indicating that faster reaction speed was associated with better performance, which is consistent with the demands of team sports. Anticipation showed a moderate positive trend (*r* = 0.34) but did not reach statistical significance (*p* = 0.08). EHC showed a small positive association (*r* = 0.18), whereas IC exhibited a small negative association with sport performance (*r* = −0.24), both of which were statistically significant. In contrast, the findings for visual-perceptual skills were limited. DP, the only visual-perceptual skill included in the meta-analysis, showed a weak and non-significant association with sport performance (*r* = 0.09, *p* = 0.56).

The associations between different visual skill domains and sport-specific performance exhibited pronounced structural differences, reflecting the multilevel organization of visual-cognitive processing in team sports. The large effect size observed for MOT may be attributable to its close correspondence with the visual information load encountered during actual match play. During competition, team-sport athletes are required to track not only the dynamic positions of the ball and opponents but also the positioning and movement of multiple teammates simultaneously (Guo et al., 2025; Zemková et al., 2025a). Previous studies (Mangine et al., 2014; Gou and Li, 2023) have consistently demonstrated that expert athletes in sports such as basketball and soccer outperform novices on MOT tasks, particularly under conditions of high target load or increased object speed. Individual differences in the capability to sustain attentional tracking in dynamic environments may directly influence passing decisions, movement judgments, and transitions between offensive and defensive phases of play (Ehmann et al., 2021; Ehmann et al., 2022). However, substantial heterogeneity was observed in the MOT analysis (*I* ^2^ > 50%). This variability may reflect differences in sport type, competitive level, age, and testing paradigms across studies. The moderate effect sizes observed for VA and VS are highly consistent with existing evidence. Team sports typically require athletes to rapidly extract task relevant information in high-speed environments involving multiple simultaneously moving players. For example, Scharfen and Memmert (2021) reported that, among Bundesliga players, selective attention and cognitive flexibility showed moderate associations with game intelligence and were predictive of playing time. In addition, studies in youth soccer (Schumacher et al., 2024) and volleyball (Trecroci et al., 2021) have demonstrated moderate associations between selective attention, visual search speed, and sport-specific motor skills, including dribbling, passing, and change-of-direction performance. These findings suggest that attentional resource allocation represents a foundational component of optimal action selection in team-sport contexts. Effective attentional allocation enables athletes to rapidly identify task relevant cues in multiagent environments and to maintain efficient visual search strategies under conditions of distraction, thereby supporting accurate technical and tactical decision-making (Lochhead et al., 2024b).

In contrast, the association effect of VWM was smaller, which is consistent with findings from multiple studies. VWM is more strongly related to the stability of complex technical execution rather than directly driving moment-to-moment decision-making during competition (Trecroci et al., 2021; Schumacher et al., 2024; Delgado et al., 2025). Therefore, the relatively smaller effect size observed for VWM may reflect its primary involvement in slower evolving processes, such as the maintenance of tactical structures and the updating of offensive and defensive patterns, which play a more indirect role in momentary performance outcomes during competition.

Among reaction time measures, the negative associations observed for SRT and CRT indicate that faster responses are associated with superior performance, in line with a substantial body of empirical evidence. For example, studies in volleyball (Trecroci et al., 2021), basketball (Zemková et al., 2025b), and soccer (Broodryk et al., 2025) consistently report that simple light based reaction time tasks show weak associations with in-game performance, whereas CRT tasks or reaction paradigms incorporating inhibition and interference processing demonstrate stronger relationships with match performance. These findings suggest that when the cognitive demands of a test more closely approximate those encountered during competition, such as directional choice and interference inhibition, the ecological validity of the task increases, resulting in stronger associations with actual competitive performance. Overall, these results indicate that the reaction speed advantage observed in high-level athletes does not simply reflect faster physiological responses, but instead reflects more efficient decision-making processes under time pressure.

A significant negative association was observed between IC and sport-specific performance. In baseball batting research (Morishita et al., 2025), showed that athletes with superior inhibitory control and faster response times performed better in pitch selection tasks. Similarly, elite soccer players demonstrate superior performance on Flanker and Stroop tasks, enabling faster suppression of irrelevant information and more efficient updating of action plans (Scharfen and Memmert, 2021). Accordingly, the negative association observed for IC likely reflects the beneficial effects of shorter inhibitory reaction times and stronger interference suppression on decision-making quality in team-sport contexts.

Although EHC showed a small positive association with sport-specific performance, its effect size was substantially weaker than that observed for other visually mediated cognitive processing skills. Previous research (Chen et al., 2021; Broodryk et al., 2025) suggests that EHC primarily supports fundamental technical actions, while showing relatively weak associations with overall match performance. As such, EHC may function more as a supportive indicator of basic motor execution efficiency rather than a direct determinant of competitive performance outcomes (Guo et al., 2024). Therefore, the small effect size observed for EHC suggests that its contribution in team sports is more closely related to the stability and consistency of technical execution, rather than to tactical choice or decision-making under high pressure conditions.

Notably, the effect associated with visual-perceptual skills, specifically DP, was substantially weaker than that observed for visual-cognitive skills. In the present analysis, DP exhibited a very small and non-significant association with sport-specific performance (*r* = 0.09, *p* = 0.56). Although early work by Isaacs (1981) reported an association between DP and free-throw shooting, such effects appear to be largely confined to static, precise aiming tasks rather than to the fast, dynamic, environments characteristic of team sport.

### 4.2 Interpretation of the findings

The present findings indicate that visual-cognitive skills show substantially stronger associations with team-sport performance than visual-perceptual skills, offering preliminary evidence for the application of the “Two-visual-system Hypothesis” to the sport domain (David et al., 2021). According to the framework proposed by David et al. (2021), human visual processing is commonly described in terms of a ventral “vision-for-perception” pathway and a dorsal “vision-for-action” pathway. The ventral pathway (Ebrahimi et al., 2024) primarily supports object and feature based processing, whereas the dorsal pathway subserves spatial localization, motion integration, and the online guidance of action. Team sports are characterized by highly dynamic and competitive environments, in which athletes are required not merely to achieve visual clarity, but to continuously judge targets, spatial relations, and action options under conditions of rapid movement. Consequently, team-sport performance places particularly high demands on dorsal pathway related capacities, including dynamic visual processing, spatial attention, and perception-action coupling.

In line with this theoretical framework, the “sports vision pyramid” (Kirschen and Laby, 2011; Lochhead et al., 2024a) provides a useful conceptualization of the hierarchical contributions of visual skills to athletic performance. Skills at the base of the pyramid predominantly rely on ventral visual processing and are typically involved in static and precise tasks. In contrast, high-level skills increasingly depend on dorsal pathway processing and more closely reflect the dynamic information processing demands encountered in real competitive environments. Accordingly, performance in team sports is more likely to be influenced by high-level skills within the pyramid than by basic visual-perceptual skills. Lochhead et al. (2024a) proposed that the visual skills most effective at discriminating between competitive levels and most strongly associated with match performance are primarily located at Levels 3 and 4 of the pyramid. These include VA, VS, MOT, and decision-making under complex conditions. In contrast, foundational functions located at the base of the pyramid, such as DP, show relatively weak associations with actual match performance and are often insufficient to account for meaningful differences in competitive performance between athletes.

Accordingly, the gradient of effect sizes observed in this study suggests that differences in the strength of associations between visual skills and sport-specific performance reflect the degree of alignment between levels of visual processing and the task demands inherent in team sports. Once athletes’ foundational visual-perceptual functions reach a functional threshold, the marginal contribution of these low-level skills to competitive performance diminishes rapidly. Under such conditions, between-athlete differences in performance are more strongly driven by high-level visual-cognitive processing (Guo et al., 2024). Visual-cognitive tasks require participants to continuously update decision strategies within dynamic environments while simultaneously imposing time pressure, information uncertainty, and distractor interference. These characteristics closely mirror the constraints inherent to team-sport competition (Appelbaum and Erickson, 2018). Therefore, the stronger associations observed with sport-specific performance are not attributable to greater task difficulty, but rather to the high degree of structural isomorphism between these tasks and real competitive situations in terms of information structure, and task demands. In other words, these high-level visual-cognitive measures more effectively capture athletes’ integrated efficiency across the full “Perception - Decision–Effector” chain (Erickson, 2021).

In addition, the variability in effect sizes across visual-cognitive indicators observed in this study highlights the heterogeneous internal structure of visual skills. Tasks such as MOT and VA, which emphasize parallel multiple object processing and spatial attentional allocation, tend to show stronger associations with tactical performance indicators, including playing time, assists, and composite efficiency metrics (Mangine et al., 2014; Scharfen and Memmert, 2021). Indicators such as VWM and IC, which reflect working memory updating and inhibitory processes, exhibit smaller overall effect sizes, yet may play a critical role in specific high-pressure decision-making scenarios. These findings indicate that visual-cognitive skill is not a unidimensional construct (Trecroci et al., 2021). High-level sport performance depends on the synergistic contribution of multiple subcomponents, with the relative weighting of these components varying substantially across sports and playing positions (Rodrigues et al., 2024b; Schumacher et al., 2024; Piechota et al., 2025). Therefore, future research should incorporate sport, position, and outcome subgroup analyses, alongside formal tests of moderating effects, to more precisely characterize these relationships.

Individual differences in visual skills, together with early developmental experiences, may predispose certain athletes to acquire performance advantages more readily during team-sport learning and to be identified earlier for entry into high-level training pathways. Conversely, prolonged exposure to high-level training and competitive environments provides frequent and highly complex perception-action coupling stimuli, which may further strengthen key capacities such as MOT, VA, and VS, thereby promoting a positive cycle of cumulative advantage. Because all included studies employed cross-sectional or observational designs, it is not possible to disentangle the relative contributions of innate predispositions and training-related adaptations. Nevertheless, the consistency and magnitude of the observed associations suggest that visual-cognitive skills constitute an important foundational source of individual differences in competitive performance.

### 4.3 Methodological considerations and quality of evidence

This study applied a random-effects model and systematically assessed heterogeneity in the associations between different visual skills and sport performance using the *I* ^2^ statistic. Overall, *I* ^2^ values for most outcomes were low, with VA, VS, VWM, EHC, IC, and SRT generally ranging between 0% and 25%. However, meaningful inconsistency was observed for several outcomes. DP and CRT demonstrated moderate heterogeneity, whereas MOT exhibited high heterogeneity (*I* ^2^ = 69%), likely reflecting variability across studies in task paradigms, measurement instruments, sport disciplines, and competitive levels. Although methodological heterogeneity was partially mitigated through the application of standardized inclusion criteria and effect-size extraction procedures, the limited number of studies available for several outcomes precluded further exploration of heterogeneity sources *via* subgroup analyses or meta-regression. Therefore, pooled effect estimates—particularly for outcomes with substantial heterogeneity such as MOT—should be interpreted with caution, and the magnitude of these effects should not be overgeneralized across different sport contexts.

It should also be acknowledged that many visual tasks inherently involve overlapping perceptual, cognitive, and motor components. For example, CRT and IC paradigms may vary substantially in cognitive load and motor execution demands across studies. Although classification was based on the predominant functional demands of each task, some conceptual overlap cannot be entirely excluded. This variability may partly contribute to observed heterogeneity and should be considered when interpreting pooled effect estimates. Although all included performance outcomes were derived from on-field assessments, the nature of these measures varied in contextual complexity. Some studies assessed isolated technical skills under structured conditions, whereas others examined competition-derived statistics reflecting multifactorial performance in dynamic environments. Differences in ecological proximity to real competitive demands may have influenced effect sizes and contributed to heterogeneity. Therefore, pooled estimates should be interpreted with consideration of these contextual distinctions.

Potential publication bias was systematically evaluated using funnel plots. Visual inspection of the funnel plots (Figure 4) did not reveal marked asymmetry for any of the included outcomes. However, with the exception of VA, fewer than 10 effect sizes were available for most outcomes. As a result, the statistical power of funnel plot assessments was limited, and more robust tests for small-study effects, such as Egger’s regression, could not be performed. Consequently, the presence of unpublished or selectively reported findings cannot be ruled out, and the magnitude or direction of the true effects may be modestly overestimated.

To assess the robustness of the pooled effect estimates, leave-one-out sensitivity analyses were conducted (Supplementary Appendix S5). The results indicated that for outcomes such as DP, SRT, MOT, and VA, the magnitude and statistical significance of the pooled correlations remained largely stable following the exclusion of any single study, indicating good robustness. However, it should be noted that several studies contributed disproportionately large weights due to substantially larger sample sizes. Consequently, exclusion of these high-weight studies resulted in more pronounced changes in the pooled effect estimates. In addition, for outcomes based on a small number of effect sizes (e.g., DP and Anticipation), as well as for MOT with substantial heterogeneity, the statistical information yielded by sensitivity analyses was limited and insufficient to fully characterize underlying uncertainty. Combined with the generally small sample sizes of the primary studies, these limitations indicate that inferences regarding these outcomes should be interpreted with caution.

The GRADE assessment varied across visual skill domains. The majority of outcomes were rated as moderate certainty, and SRT was graded as high certainty. However, Anticipation and MOT were downgraded to low certainty due to imprecision and heterogeneity. These ratings reflect variations in sample size, study design, and methodological limitations across included studies.

Furthermore, the restriction to English-language publications may have introduced language bias and potentially excluded relevant studies published in other languages, which could affect the comprehensiveness of the evidence synthesis.

### 4.4 Future directions

Future research may be advanced along several key directions: (1) All studies included in this review relied on cross-sectional or observational designs. Although such designs are informative for identifying association patterns between visual skills and sport-specific performance, they preclude causal inference. Future research should use longitudinal designs with repeated assessments to examine the temporal sequencing between visual skill development and subsequent changes in sport performance. (2) Team sports differ markedly in tactical structure, informational load, and decision-making complexity, and visual-cognitive demands also vary substantially across playing positions within the same sport. Many existing studies pool athletes across positions, thereby obscuring meaningful sources of heterogeneity. Future research should implement sport and position analyses to delineate the relative contribution of different visual skills across contexts, potentially informing more individualized approaches to athlete development. (3) Visual information processing during real competition is shaped by multiple interacting factors, including physical fatigue, psychological stress, and opponent interference, and requires decision-making under dynamic and uncertain conditions. Future research should develop assessment paradigms with greater ecological validity and integrate visual testing with neurophysiological measures to elucidate the neural mechanisms underlying visuomotor control in sport. (4) Visual skills do not operate in isolation, but interact dynamically with physical capacities, technical-tactical proficiency, and psychological characteristics. Future research may use machine learning, network analysis, and other data driven approaches to develop integrated predictive models, thereby identifying key performance pathways and interaction effects among multiple performance determinants.

## 5 Conclusion

This systematic review and meta-analysisexamined the associations between visual skills and sport-specific performance in team sports. The results suggest meaningful associations between visual–cognitive skills and sport-specific performance. MOT demonstrated a large effect size, VA and VS showed moderate effect sizes, whereas VWM and IC exhibited small effects. Reaction time measures were moderately and negatively associated with performance, highlighting the importance of visual-cognitive skills in team-sport performance. Future research should use standardized assessment protocols and ecologically valid paradigms to further clarify the role of visual skills in competitive contexts.

## Data Availability

The original contributions presented in the study are included in the article/Supplementary Material, further inquiries can be directed to the corresponding author.
