# Supplementary material for: Exploring the association between visual skills and sport-specific performance in team athletes: a systematic review and meta-analysis
Source: Front Physiol. 2026 Mar 30;17:1797347. doi: 10.3389/fphys.2026.1797347 (PMC13070756; doi:10.3389/fphys.2026.1797347)
Supplement: Supplementary file 1 [file Supplementaryfile1.docx]

**Supplementary Appendices**

**Title**

Exploring the association between visual skills and sport-specific performance in team athletes: a systematic review and meta-analysis

**Author List**

Mulin Yang^1†^, Yuqiang Guo^2†^, Feng Yang^3^, Kewei Zhao^1*^

**Affiliations**

^1^ Physical Fitness Training Research Center, China Institute of Sport Science

^2^ School of Physical Education, Shanghai University of Sport

^3^ Graduate School, Shandong Sport University

**Context**

[Appendix S1 – RISMA 2020 Checklist 1](#_Toc224893305)

[Appendix S2 – Search Strategy 5](#_Toc224893306)

[Appendix S3 – Forest plot 7](#_Toc224893307)

[Appendix S4 – Funnel plot 10](#_Toc224893308)

[Appendix S5 – Sensitivity analysis 15](#_Toc224893309)

# Appendix S1 – RISMA 2020 Checklist

| **Section and Topic** | **Item #** | **Checklist item** | **Location where item is reported** |
| --- | --- | --- | --- |
| **TITLE** | | |  |
| Title | 1 | Identify the report as a systematic review. | Page 01 |
| **ABSTRACT** | | |  |
| Abstract | 2 | See the PRISMA 2020 for Abstracts checklist. | Page 01 |
| **INTRODUCTION** | | |  |
| Rationale | 3 | Describe the rationale for the review in the context of existing knowledge. | Page 02 |
| Objectives | 4 | Provide an explicit statement of the objective(s) or question(s) the review addresses. | Page 02 |
| **METHODS** | | |  |
| Eligibility criteria | 5 | Specify the inclusion and exclusion criteria for the review and how studies were grouped for the syntheses. | Page 04-05 |
| Information sources | 6 | Specify all databases, registers, websites, organisations, reference lists and other sources searched or consulted to identify studies. Specify the date when each source was last searched or consulted. | Page 02, 04 |
| Search strategy | 7 | Present the full search strategies for all databases, registers and websites, including any filters and limits used. | Appendix S2 |
| Selection process | 8 | Specify the methods used to decide whether a study met the inclusion criteria of the review, including how many reviewers screened each record and each report retrieved, whether they worked independently, and if applicable, details of automation tools used in the process. | Page 05 |
| Data collection process | 9 | Specify the methods used to collect data from reports, including how many reviewers collected data from each report, whether they worked independently, any processes for obtaining or confirming data from study investigators, and if applicable, details of automation tools used in the process. | Page 05-06 |
| –-Data items | 10a | List and define all outcomes for which data were sought. Specify whether all results that were compatible with each outcome domain in each study were sought (e.g. for all measures, time points, analyses), and if not, the methods used to decide which results to collect. | Page 05 |
|  | 10b | List and define all other variables for which data were sought (e.g. participant and intervention characteristics, funding sources). Describe any assumptions made about any missing or unclear information. | Page 05 |
| Study risk of bias assessment | 11 | Specify the methods used to assess risk of bias in the included studies, including details of the tool(s) used, how many reviewers assessed each study and whether they worked independently, and if applicable, details of automation tools used in the process. | Page 06 |
| Effect measures | 12 | Specify for each outcome the effect measure(s) (e.g. risk ratio, mean difference) used in the synthesis or presentation of results. | Page 06 |
| Synthesis methods | 13a | Describe the processes used to decide which studies were eligible for each synthesis (e.g. tabulating the study intervention characteristics and comparing against the planned groups for each synthesis (item #5)). | Table 1 |
|  | 13b | Describe any methods required to prepare the data for presentation or synthesis, such as handling of missing summary statistics, or data conversions. | Page 06 |
|  | 13c | Describe any methods used to tabulate or visually display results of individual studies and syntheses. | Page 06 |
|  | 13d | Describe any methods used to synthesize results and provide a rationale for the choice(s). If meta-analysis was performed, describe the model(s), method(s) to identify the presence and extent of statistical heterogeneity, and software package(s) used. | Page 06 |
|  | 13e | Describe any methods used to explore possible causes of heterogeneity among study results (e.g. subgroup analysis, meta-regression). | Page 06 |
|  | 13f | Describe any sensitivity analyses conducted to assess robustness of the synthesized results. | Page 06-07 |
| Reporting bias assessment | 14 | Describe any methods used to assess risk of bias due to missing results in a synthesis (arising from reporting biases). | Page 06 |
| Certainty assessment | 15 | Describe any methods used to assess certainty (or confidence) in the body of evidence for an outcome. | Page 06 |
| **RESULTS** | | |  |
| Study selection | 16a | Describe the results of the search and selection process, from the number of records identified in the search to the number of studies included in the review, ideally using a flow diagram. | Page 07 |
|  | 16b | Cite studies that might appear to meet the inclusion criteria, but which were excluded, and explain why they were excluded. | / |
| Study characteristics | 17 | Cite each included study and present its characteristics. | Table 1 |
| Risk of bias in studies | 18 | Present assessments of risk of bias for each included study. | Figure 2 & 3 |
| Results of individual studies | 19 | For all outcomes, present, for each study: (a) summary statistics for each group (where appropriate) and (b) an effect estimate and its precision (e.g. confidence/credible interval), ideally using structured tables or plots. | Figure 07 |
| Results of syntheses | 20a | For each synthesis, briefly summarise the characteristics and risk of bias among contributing studies. | Page 07 |
|  | 20b | Present results of all statistical syntheses conducted. If meta-analysis was done, present for each the summary estimate and its precision (e.g. confidence/credible interval) and measures of statistical heterogeneity. If comparing groups, describe the direction of the effect. | Page 07-10,  Figure 4 |
|  | 20c | Present results of all investigations of possible causes of heterogeneity among study results. | Page 07-10, Figure 4 |
|  | 20d | Present results of all sensitivity analyses conducted to assess the robustness of the synthesized results. | Page 07-10  Appendix S5 |
| Reporting biases | 21 | Present assessments of risk of bias due to missing results (arising from reporting biases) for each synthesis assessed. | Page 07 |
| Certainty of evidence | 22 | Present assessments of certainty (or confidence) in the body of evidence for each outcome assessed. | Figure 4 |
| **DISCUSSION** | | |  |
| Discussion | 23a | Provide a general interpretation of the results in the context of other evidence. | Page 10 |
|  | 23b | Discuss any limitations of the evidence included in the review. | Page 12 |
|  | 23c | Discuss any limitations of the review processes used. | Page 12 |
|  | 23d | Discuss implications of the results for practice, policy, and future research. | Page 12 |
| **OTHER INFORMATION** | | |  |
| Registration and protocol | 24a | Provide registration information for the review, including register name and registration number, or state that the review was not registered. | Page 02 |
|  | 24b | Indicate where the review protocol can be accessed, or state that a protocol was not prepared. | Page 02 |
|  | 24c | Describe and explain any amendments to information provided at registration or in the protocol. | / |
| Support | 25 | Describe sources of financial or non-financial support for the review, and the role of the funders or sponsors in the review. | Page 13 |
| Competing interests | 26 | Declare any competing interests of review authors. | Page 13 |
| Availability of data, code and other materials | 27 | Report which of the following are publicly available and where they can be found: template data collection forms; data extracted from included studies; data used for all analyses; analytic code; any other materials used in the review. | Page 13 |

# Appendix S2 – Search Strategy

| ***Web of Science (Core Collection) (n = 1366)***  TS=("visual skill*" OR "visual abilit*" OR "visual function*" OR "visual performance" OR "vision skill*" OR "visual capacit*" OR "visual acuity" OR "depth perception" OR "peripheral vision" OR "eye-hand coordination" OR "visual tracking" OR "dynamic visual acuity" OR "contrast sensitivity" OR "reaction time" OR "visual motor*" OR "visuomotor" OR "ocular motor*" OR "eye movement*" OR "visual perception" OR "visual processing" OR "sports vision" OR "visual attention" OR "multiple object tracking" OR "MOT") AND TS=("team sport*" OR "team athlete*" OR "team player*" OR "team-based sport*" OR "collective sport*" OR soccer OR football OR basketball OR volleyball OR handball OR hockey OR "ice hockey" OR "field hockey" OR rugby OR "water polo" OR baseball OR softball OR cricket OR lacrosse OR netball OR "team game*" OR "invasion game*" OR "ball sport*") |
| --- |
| ***PubMed (n = 1110)***  #1 "visual skill*"[Title/Abstract] OR "visual abilit*"[Title/Abstract] OR "visual function*"[Title/Abstract] OR "visual performance"[Title/Abstract] OR "vision skill*"[Title/Abstract] OR "visual capacit*"[Title/Abstract] OR "visual acuity"[Title/Abstract] OR "depth perception"[Title/Abstract] OR "peripheral vision"[Title/Abstract] OR "eye-hand coordination"[Title/Abstract] OR "visual tracking"[Title/Abstract] OR "dynamic visual acuity"[Title/Abstract] OR "contrast sensitivity"[Title/Abstract] OR "reaction time"[Title/Abstract] OR "visual motor*"[Title/Abstract] OR "visuomotor"[Title/Abstract] OR "ocular motor*"[Title/Abstract] OR "eye movement*"[Title/Abstract] OR "visual perception"[Title/Abstract] OR "visual processing"[Title/Abstract] OR "sports vision"[Title/Abstract] OR "visual attention"[Title/Abstract] OR "multiple object tracking"[Title/Abstract] OR "MOT"[Title/Abstract]  #2 "team sport*"[Title/Abstract] OR "team athlete*"[Title/Abstract] OR "team player*"[Title/Abstract] OR "team-based sport*"[Title/Abstract] OR "collective sport*"[Title/Abstract] OR soccer[Title/Abstract] OR football[Title/Abstract] OR basketball[Title/Abstract] OR volleyball[Title/Abstract] OR handball[Title/Abstract] OR hockey[Title/Abstract] OR "ice hockey"[Title/Abstract] OR "field hockey"[Title/Abstract] OR rugby[Title/Abstract] OR "water polo"[Title/Abstract] OR baseball[Title/Abstract] OR softball[Title/Abstract] OR cricket[Title/Abstract] OR lacrosse[Title/Abstract] OR netball[Title/Abstract] OR "team game*"[Title/Abstract] OR "invasion game*"[Title/Abstract] OR "ball sport*"[Title/Abstract]  #3 #1 AND #2 |
| ***MEDLINE (n = 1007) and SPORTDiscus (n = 637) through EBSCOhost***  XB ("visual skill*" OR "visual abilit*" OR "visual function*" OR "visual performance" OR "vision skill*" OR "visual capacit*" OR "visual acuity" OR "depth perception" OR "peripheral vision" OR "eye-hand coordination" OR "visual tracking" OR "dynamic visual acuity" OR "contrast sensitivity" OR "reaction time" OR "visual motor*" OR "visuomotor" OR "ocular motor*" OR "eye movement*" OR "visual perception" OR "visual processing" OR "sports vision" OR "visual attention" OR "multiple object tracking" OR "MOT") AND XB ("team sport*" OR "team athlete*" OR "team player*" OR "team-based sport*" OR "collective sport*" OR soccer OR football OR basketball OR volleyball OR handball OR hockey OR "ice hockey" OR "field hockey" OR rugby OR "water polo" OR baseball OR softball OR cricket OR lacrosse OR netball OR "team game*" OR "invasion game*" OR "ball sport*") |

*Note:*

*The systematic literature search included studies published up to October 20, 2025.*

# Appendix S3 – Forest plot


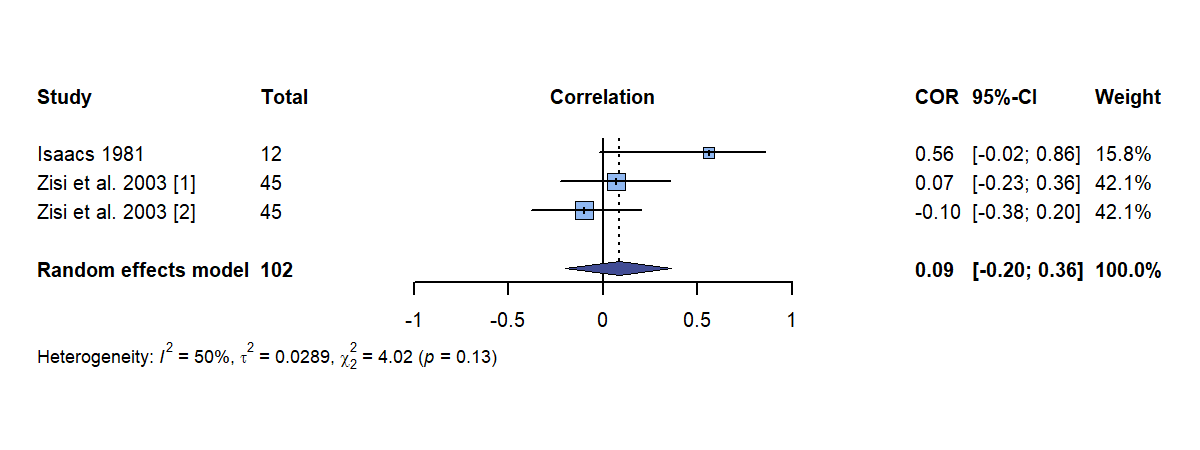


Figure 3.1 Forest plot of the association between **Depth Perception** and sport-specific performance


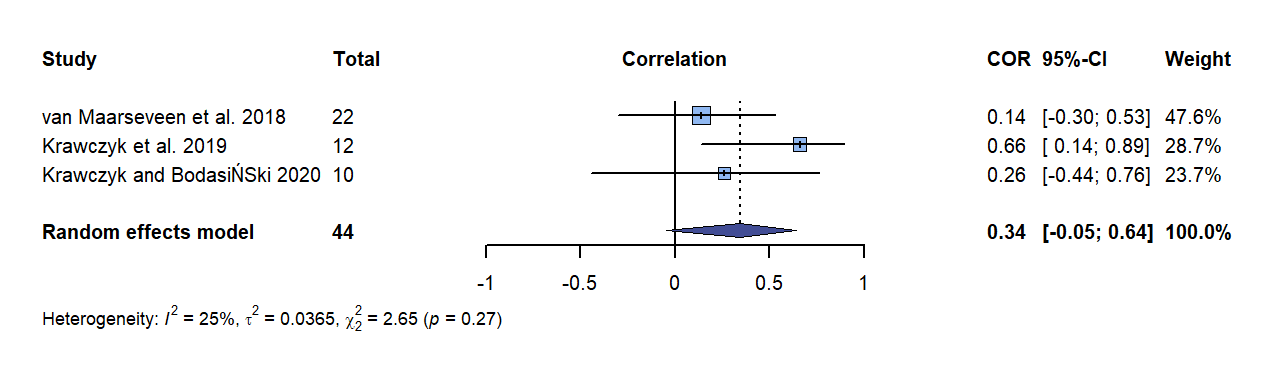


Figure 3.2 Forest plot of the association between **Anticipation** and sport-specific performance


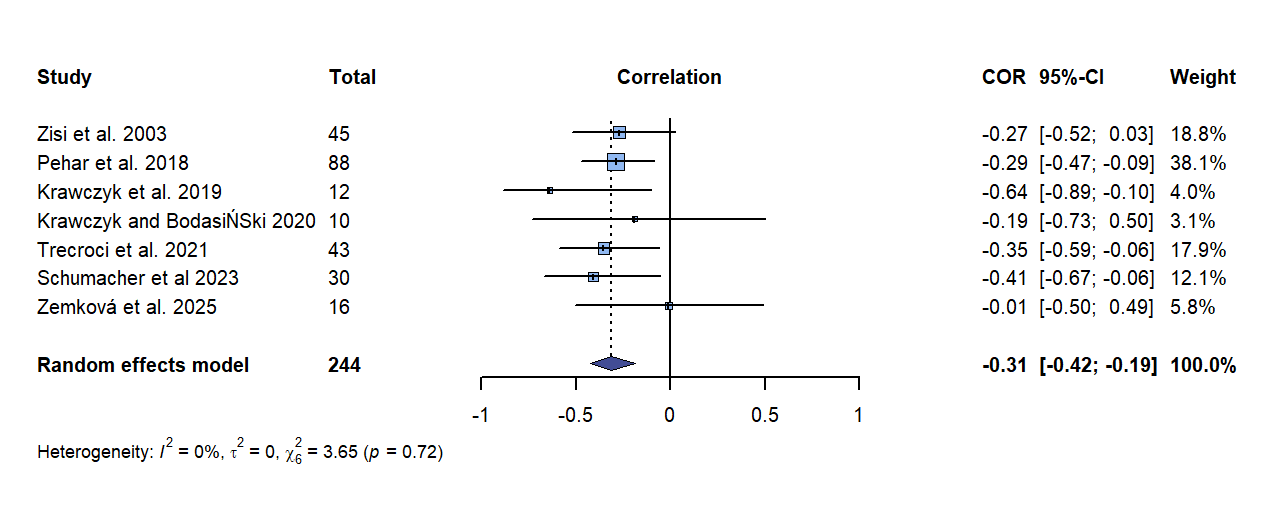


Figure 3.3 Forest plot of the association between **Simple Reaction Time** and sport-specific performance


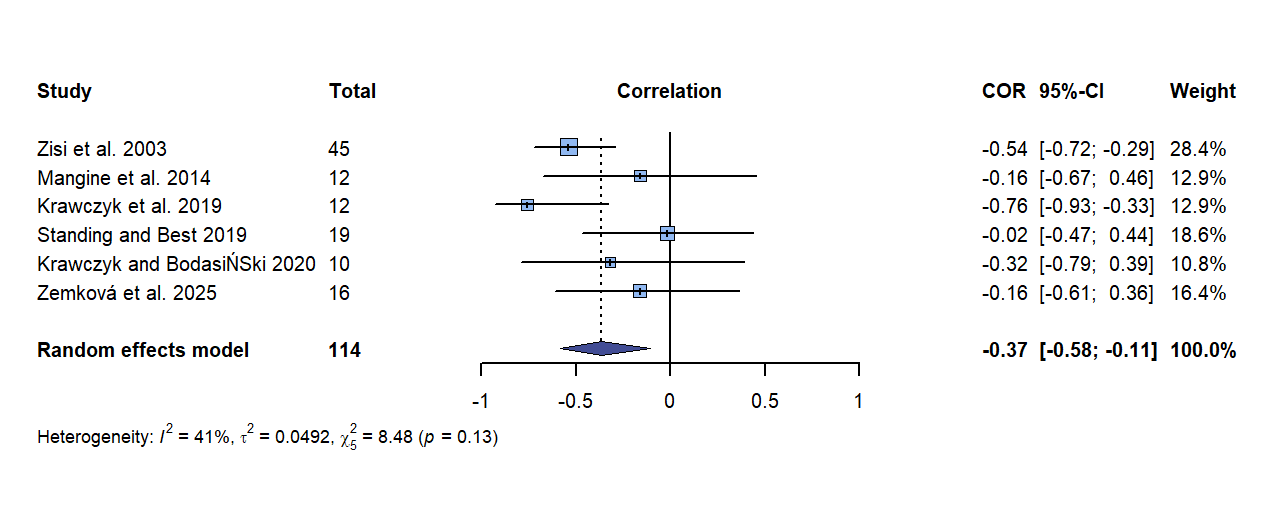


Figure 3.4 Forest plot of the association between **Choice Reaction Time** and sport-specific performance

**
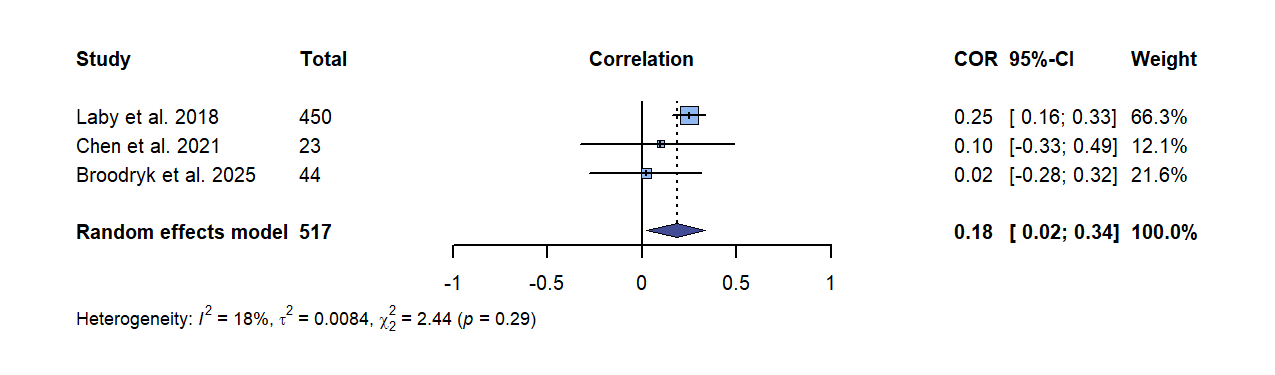
**

Figure 3.5 Forest plot of the association between **Eye-hand Coordination** and sport-specific performance

**
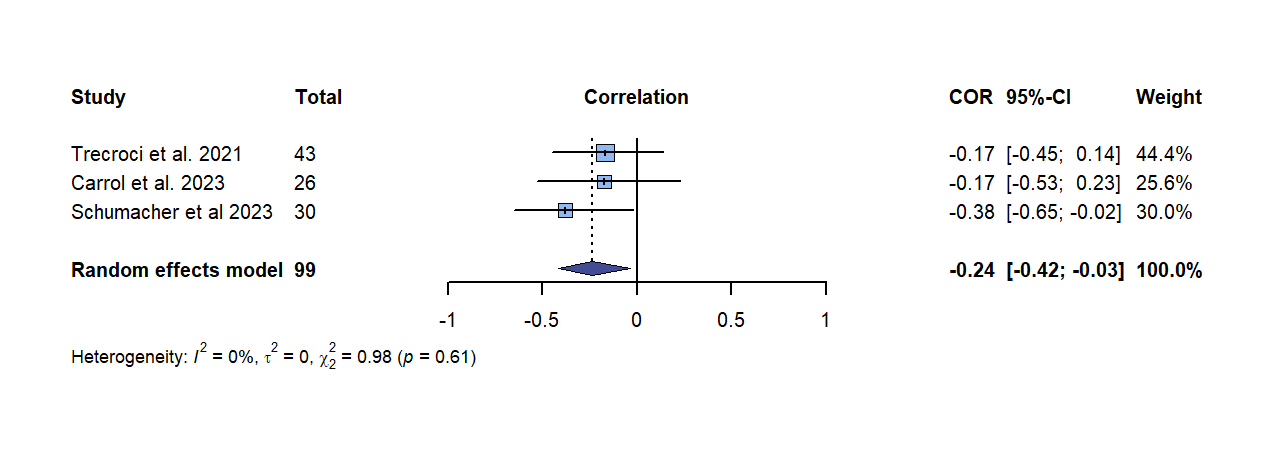
**

Figure 3.6 Forest plot of the association between **Inhibitory Control** and sport-specific performance

**
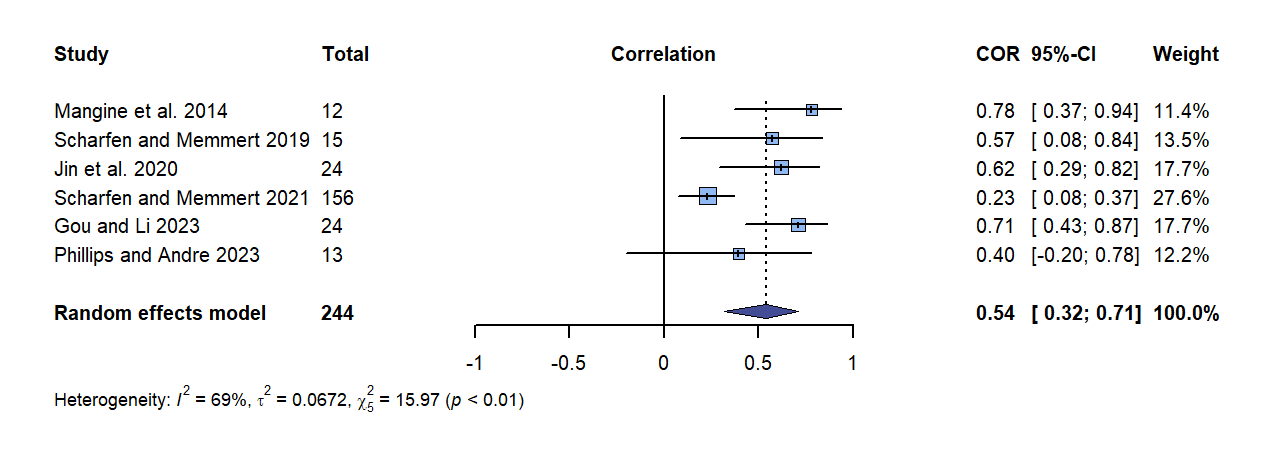
**

Figure 3.7 Forest plot of the association between **Multiple Object Tracking** and sport-specific performance

**
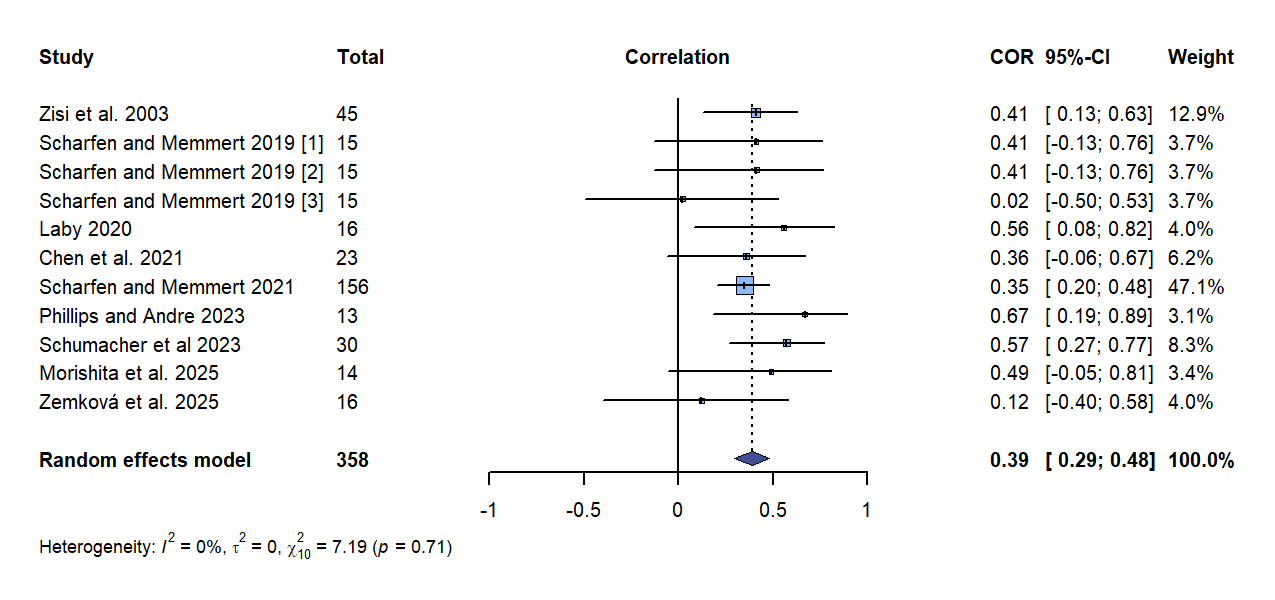
**

Figure 3.8 Forest plot of the association between **Visual Attention** and sport-specific performance


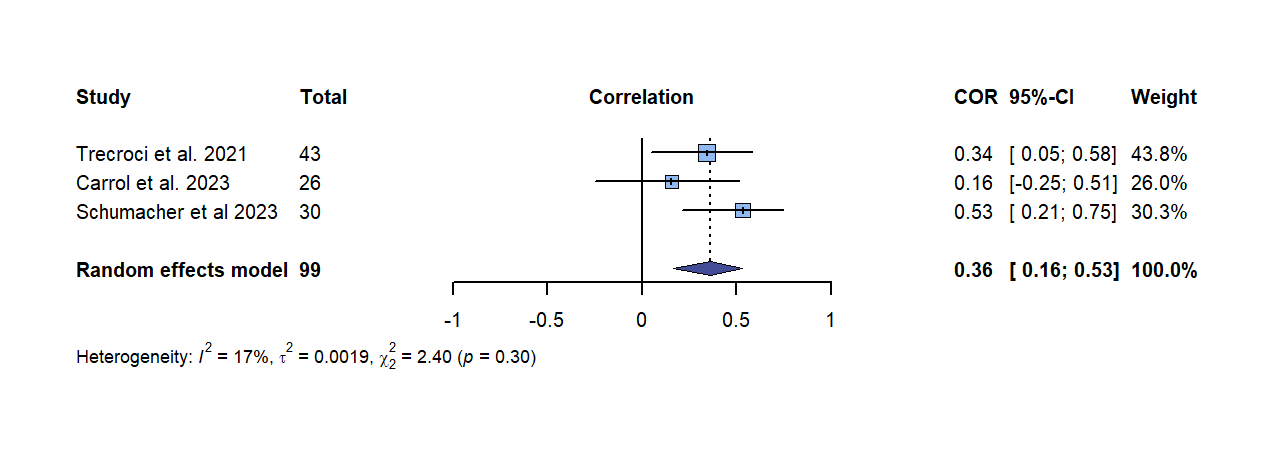


Figure 3.9 Forest plot of the association between **Visual Search** and sport-specific performance


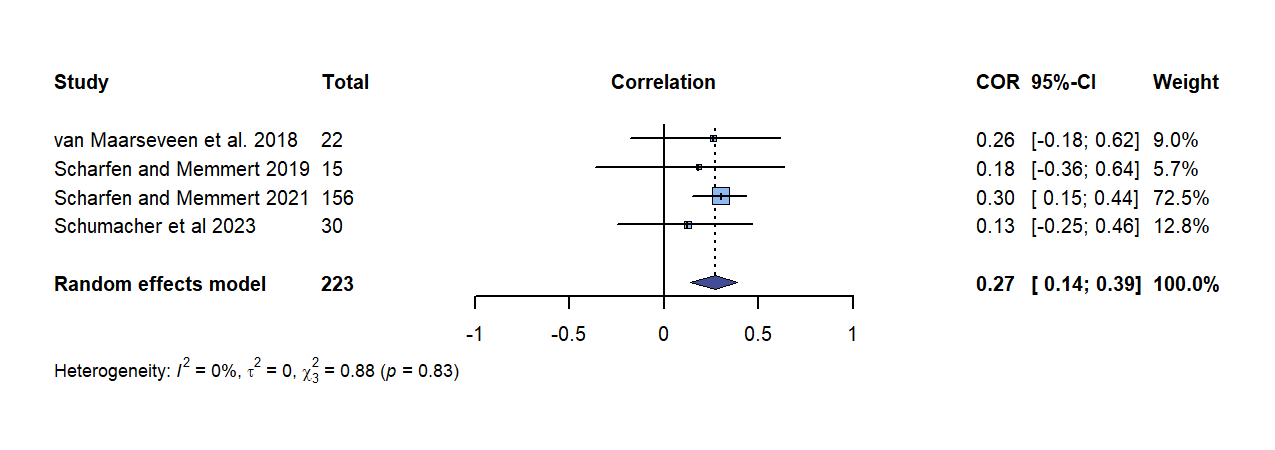


Figure 3.10 Forest plot of the association between **Visual Working Memory** and sport-specific performance

# Appendix S4 – Funnel plot


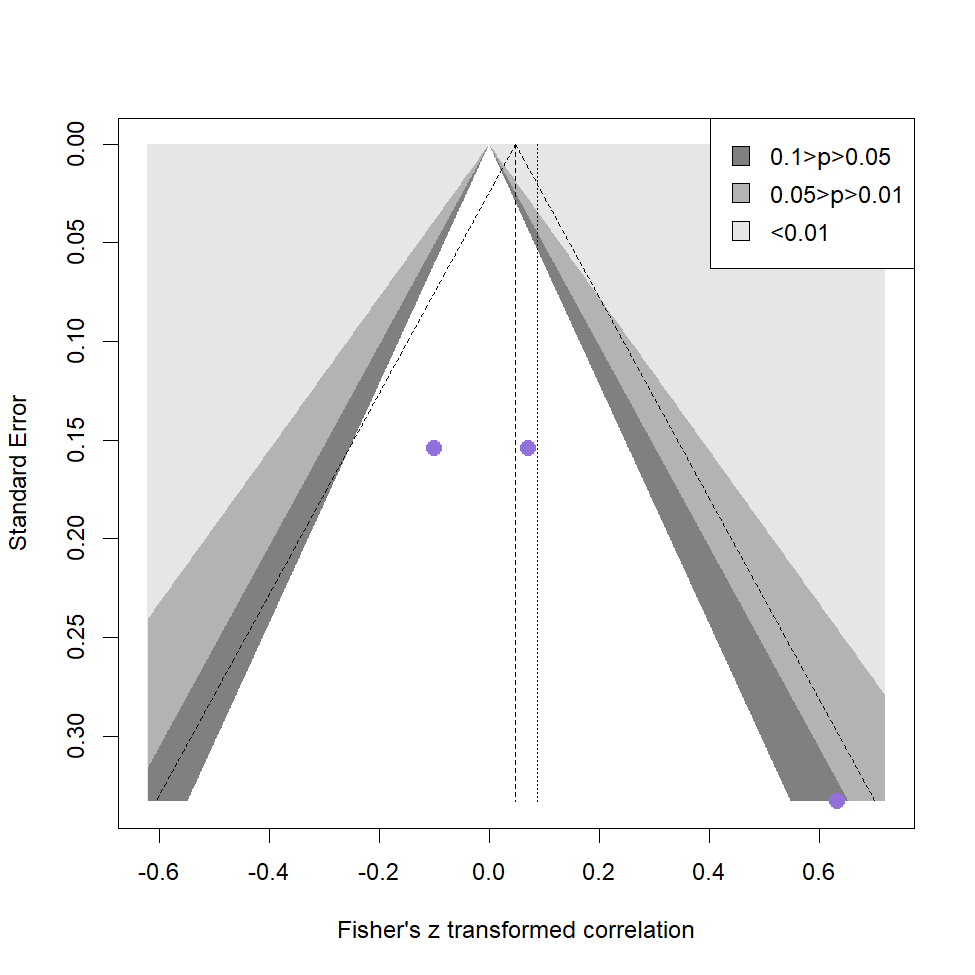


Figure 4.1 Funnel plot assessing potential publication bias for studies examining the association between **Depth Perception** and sport-specific performance


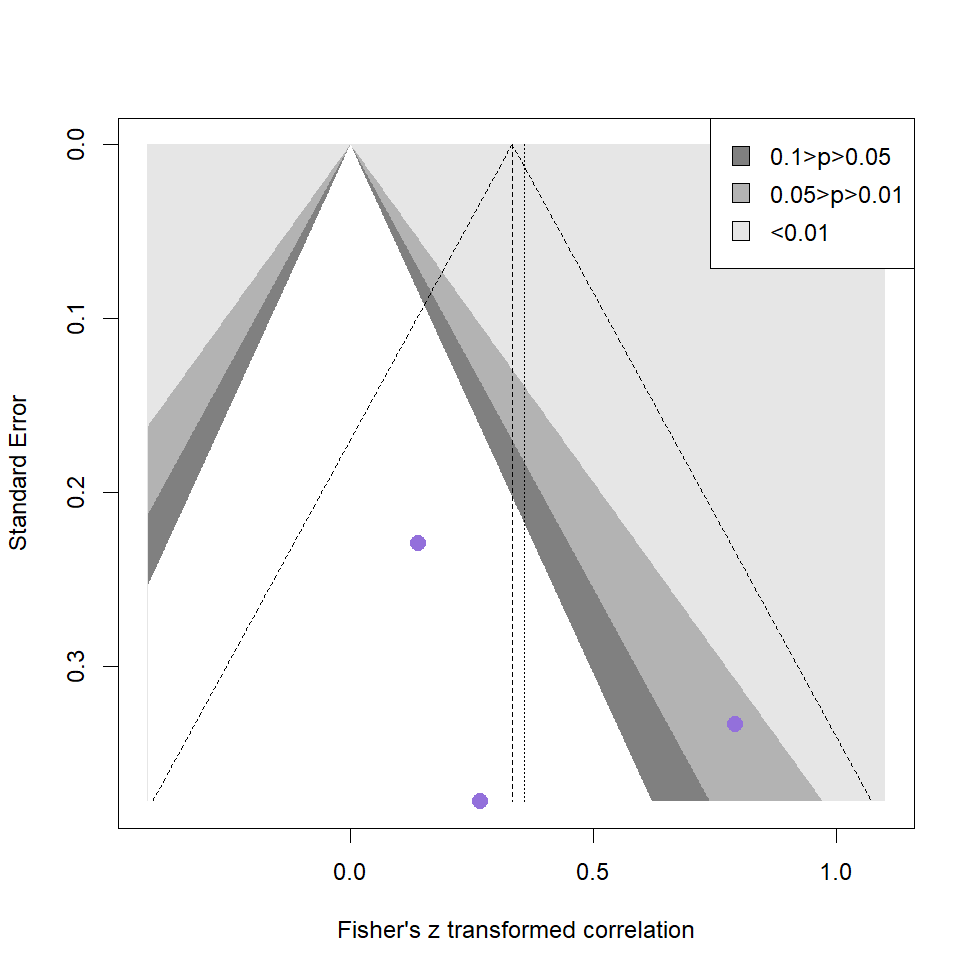


Figure 4.2 Funnel plot assessing potential publication bias for studies examining the association between **Anticipation** and sport-specific performance


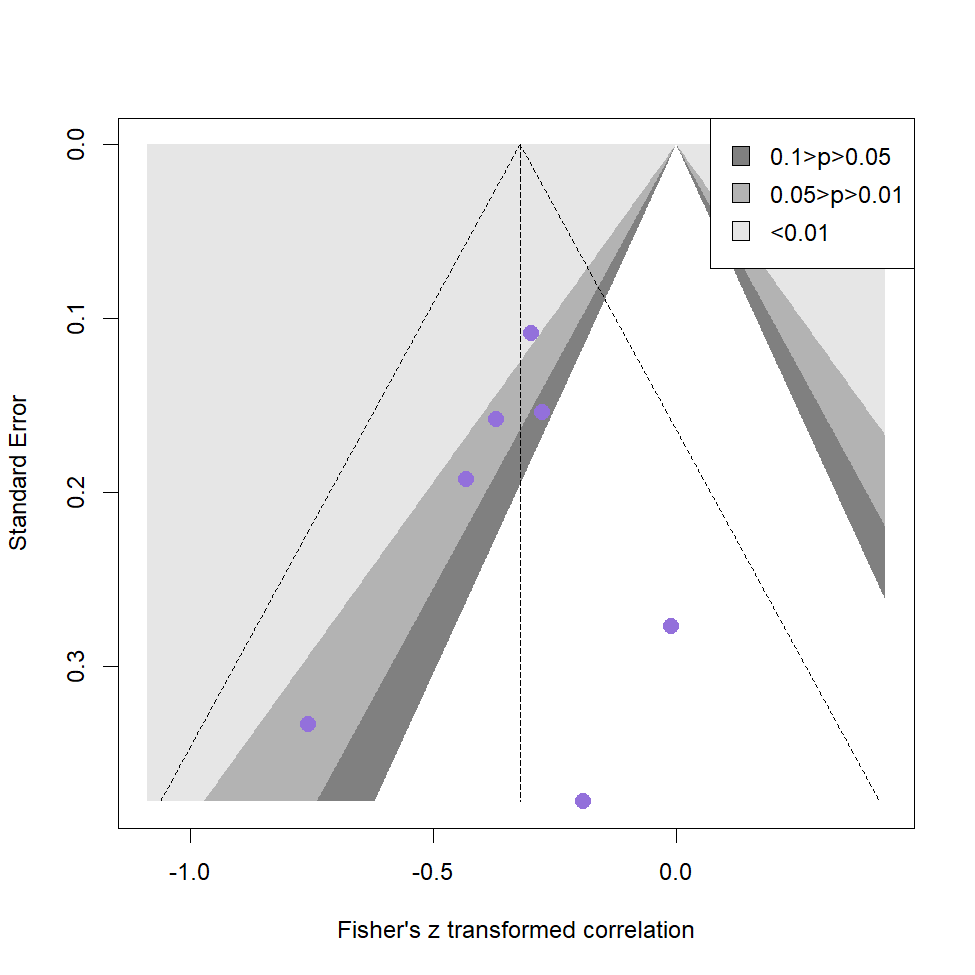


Figure 4.3 Funnel plot assessing potential publication bias for studies examining the association between **Simple Reaction Time** and sport-specific performance


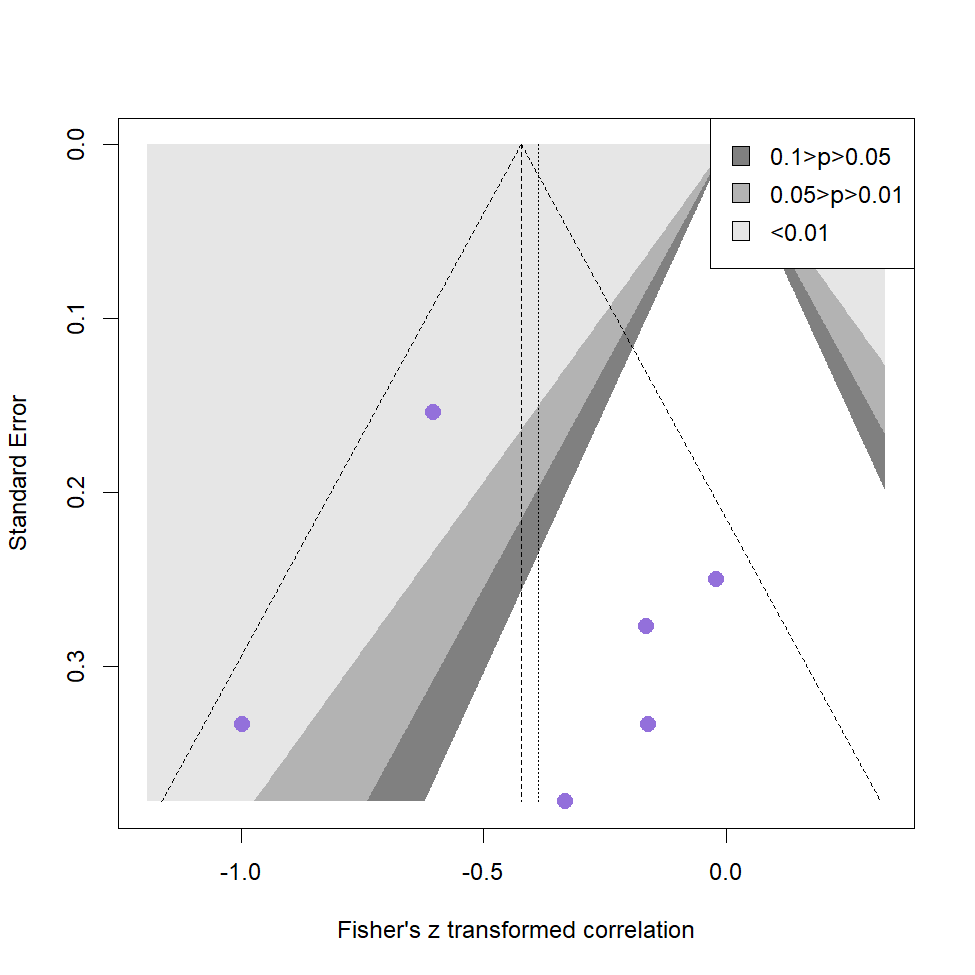


Figure 4.4 Funnel plot assessing potential publication bias for studies examining the association between **Choice Reaction Time** and sport-specific performance

**
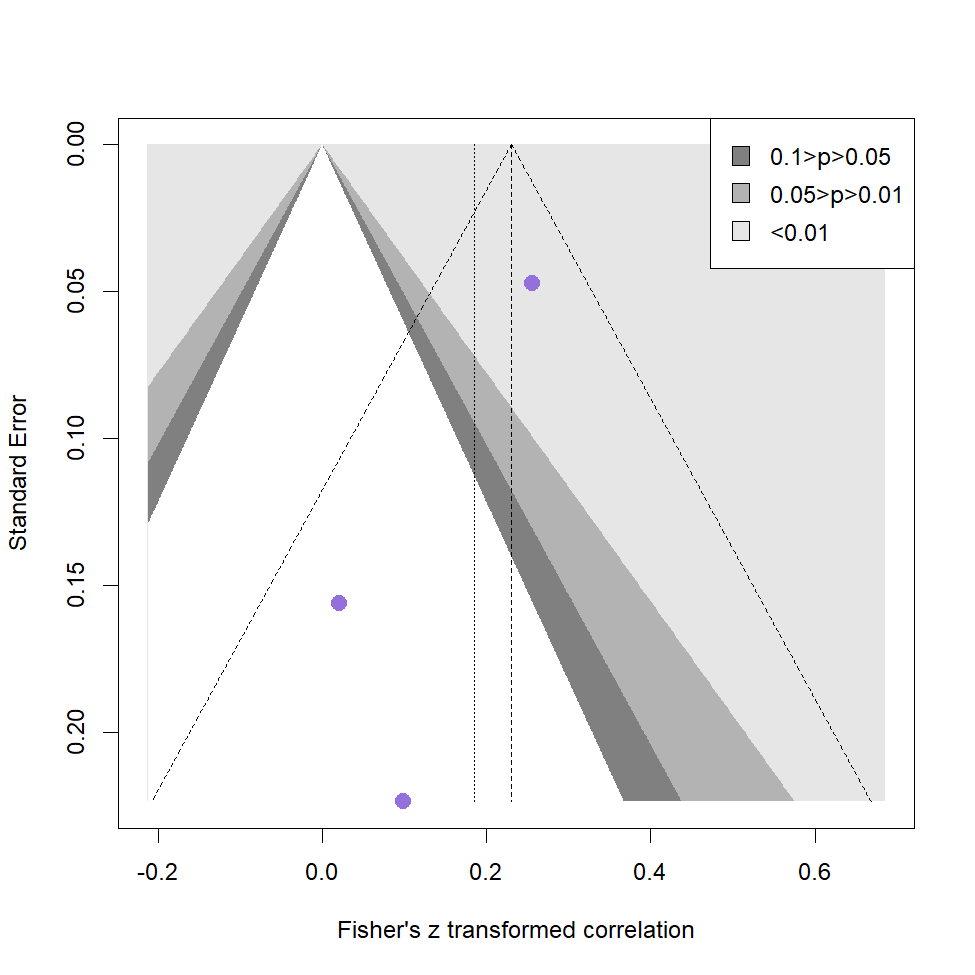
**

Figure 4.5 Funnel plot assessing potential publication bias for studies examining the association between **Eye-hand Coordination** and sport-specific performance

**
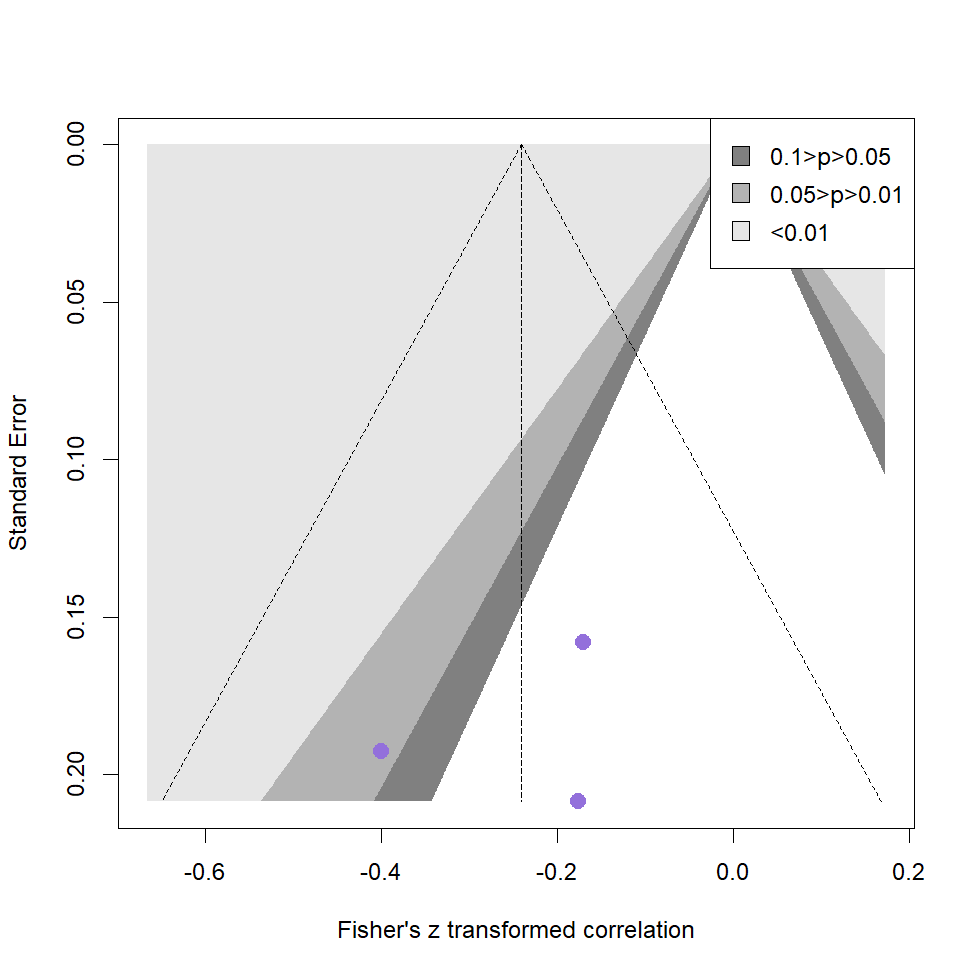
**

Figure 4.6 Funnel plot assessing potential publication bias for studies examining the association between **Inhibitory Control** and sport-specific performance

**
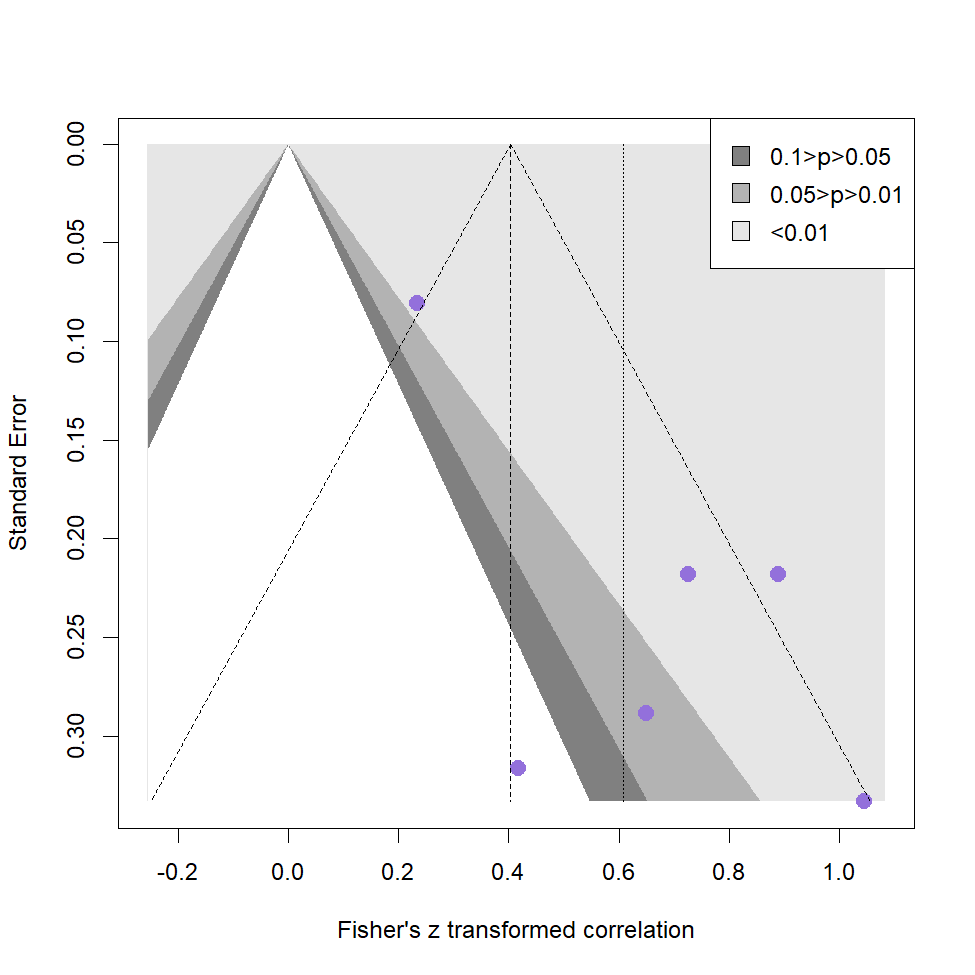
**

Figure 4.7 Funnel plot assessing potential publication bias for studies examining the association between **Multiple Object Tracking** and sport-specific performance

**
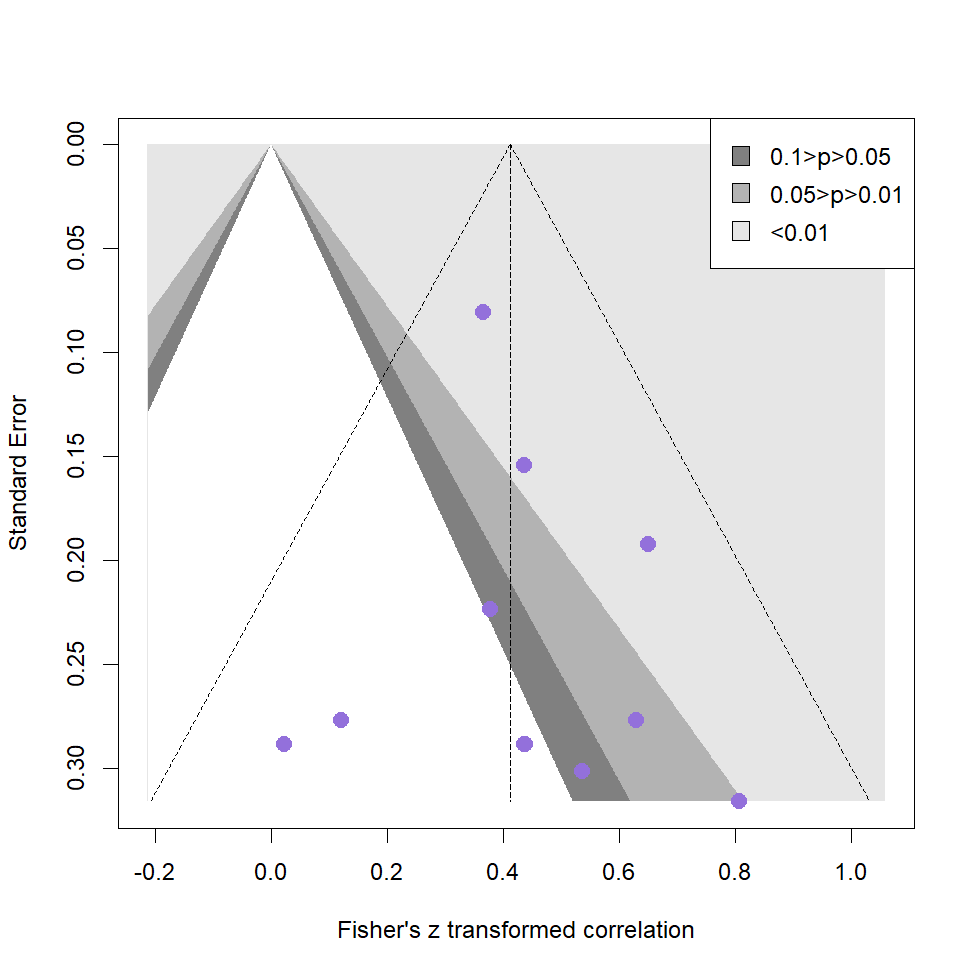
**

Figure 4.8 Funnel plot assessing potential publication bias for studies examining the association between **Visual Attention** and sport-specific performance


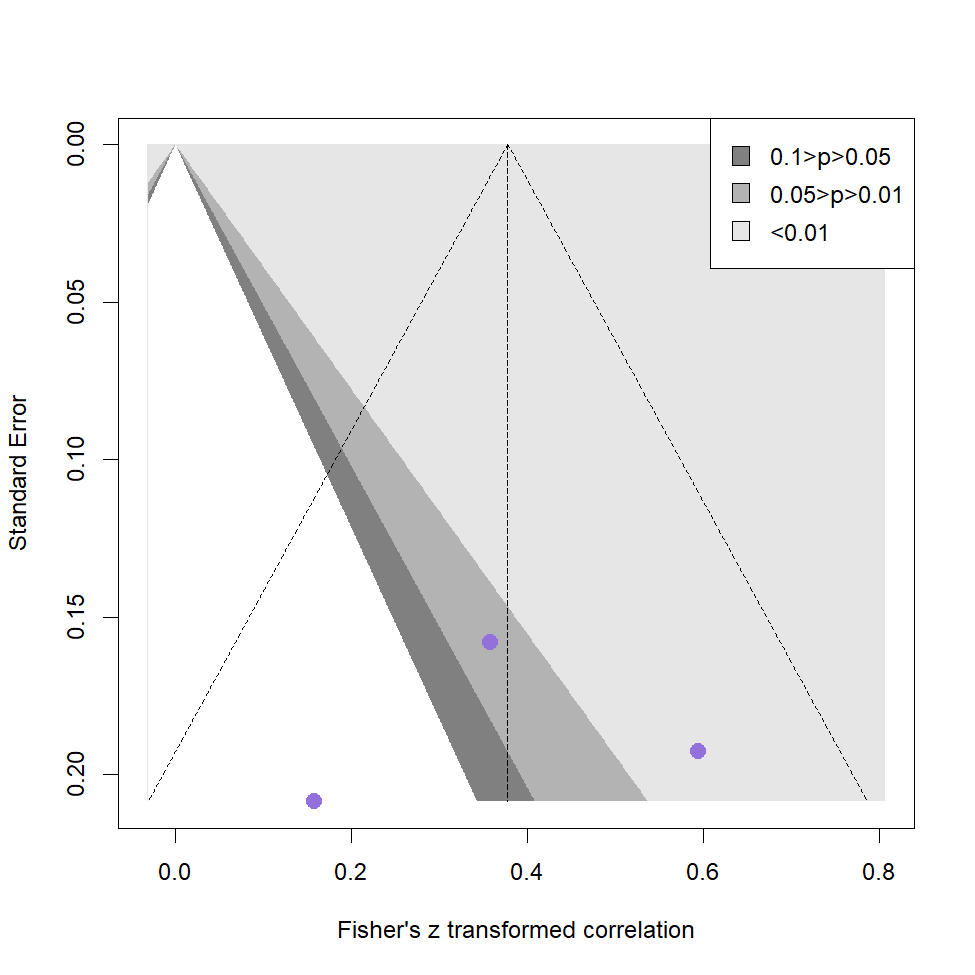


Figure 4.9 Funnel plot assessing potential publication bias for studies examining the association between **Visual Search** and sport-specific performance


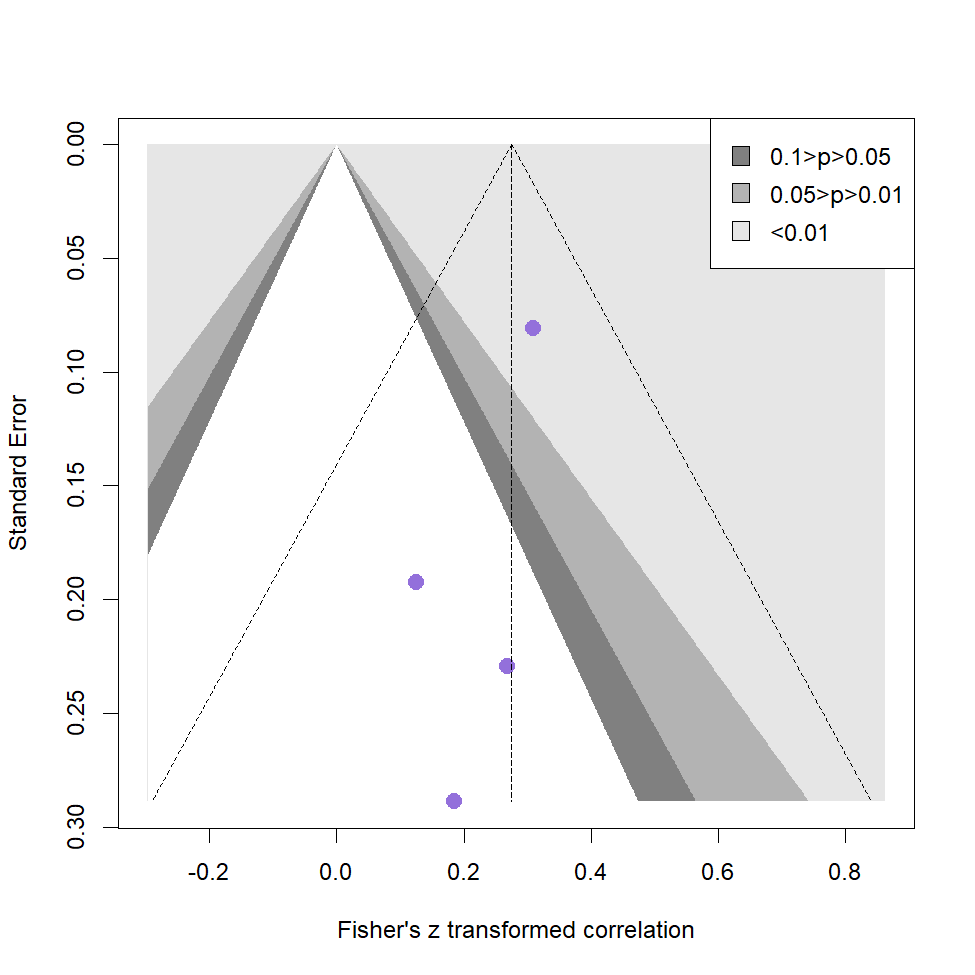


Figure 4.10 Funnel plot assessing potential publication bias for studies examining the association between **Visual Working Memory** and sport-specific performance

# Appendix S5 – Sensitivity analysis


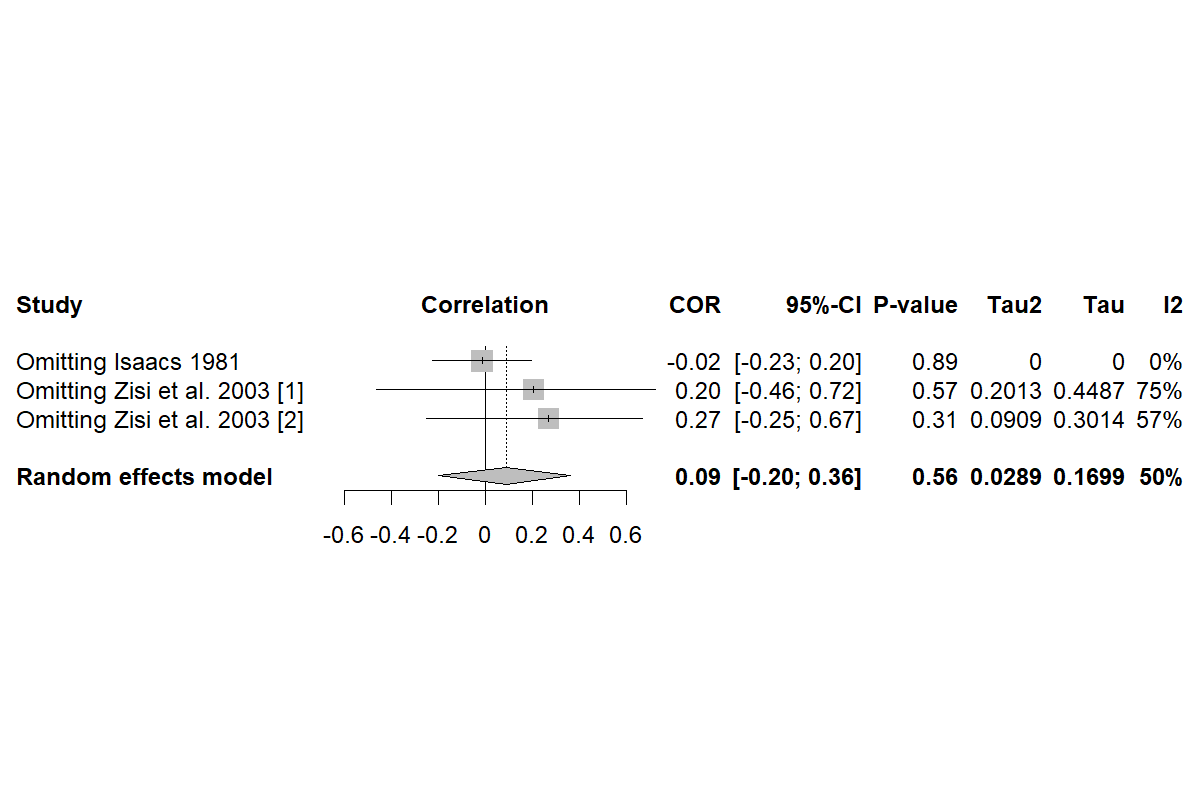


Figure 5.1 Sensitivity analysis (leave-one-out) of the pooled correlation between **Depth Perception** and sport-specific performance using a random-effects model


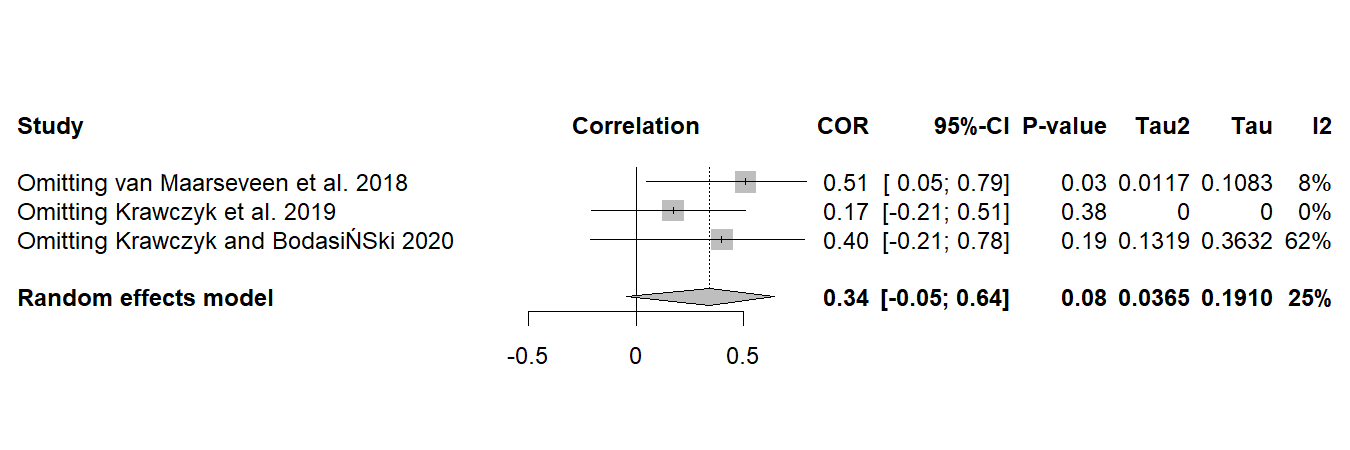


Figure 5.2 Sensitivity analysis (leave-one-out) of the pooled correlation between **Anticipation** and sport-specific performance using a random-effects model


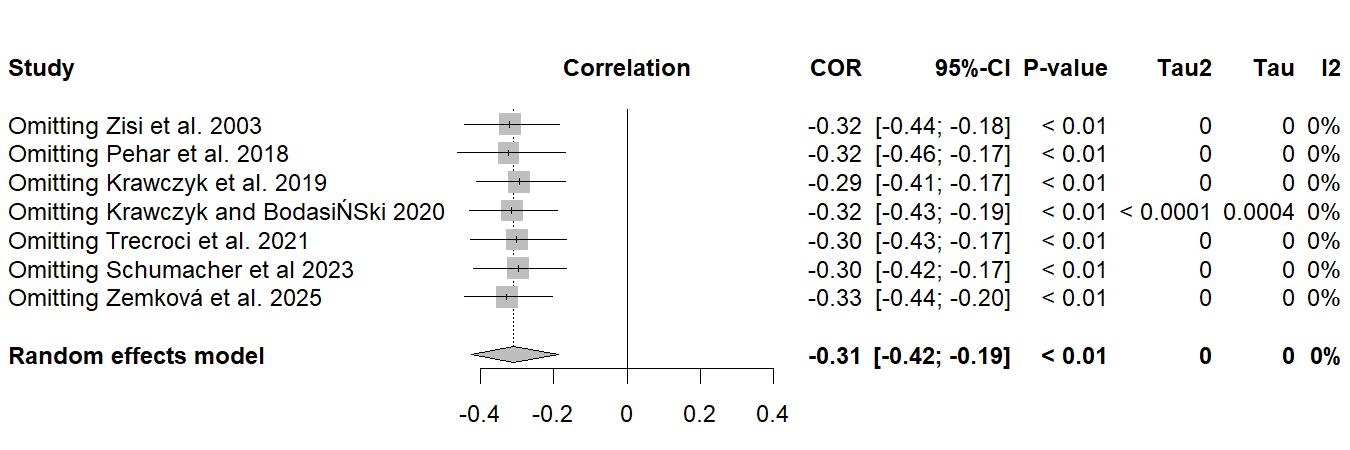


Figure 5.3 Sensitivity analysis (leave-one-out) of the pooled correlation between **Simple Reaction Time** and sport-specific performance using a random-effects model


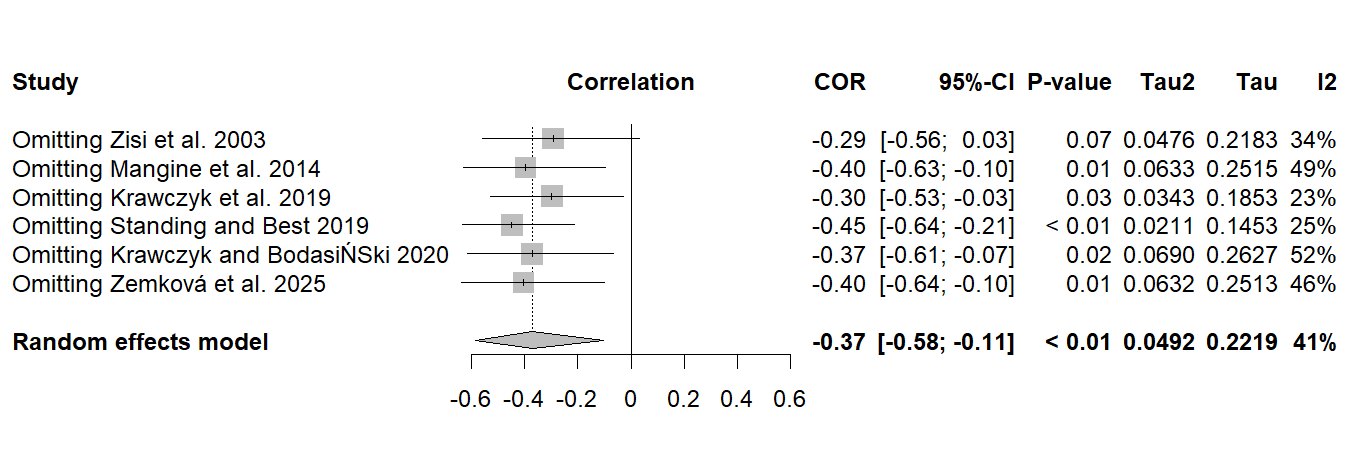


Figure 5.4 Sensitivity analysis (leave-one-out) of the pooled correlation between **Choice Reaction Time** and sport-specific performance using a random-effects model

**
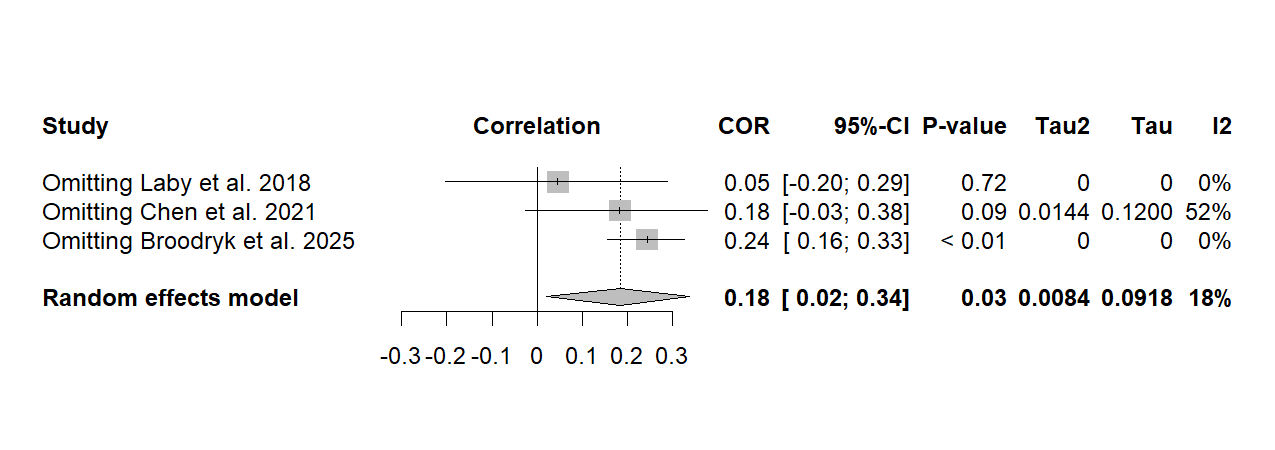
**

Figure 5.5 Sensitivity analysis (leave-one-out) of the pooled correlation between **Eye-hand Coordination** and sport-specific performance using a random-effects model

**
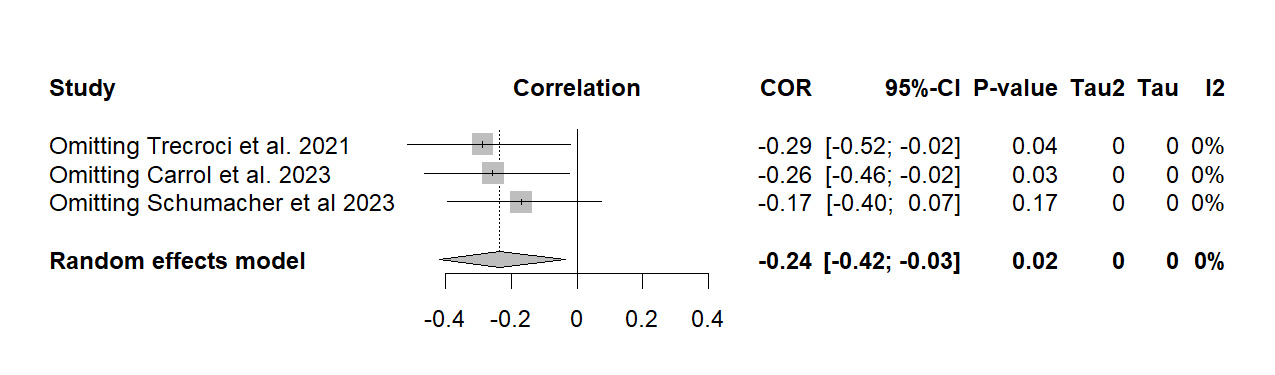
**

Figure 5.6 Sensitivity analysis (leave-one-out) of the pooled correlation between **Inhibitory Control** and sport-specific performance using a random-effects model

**
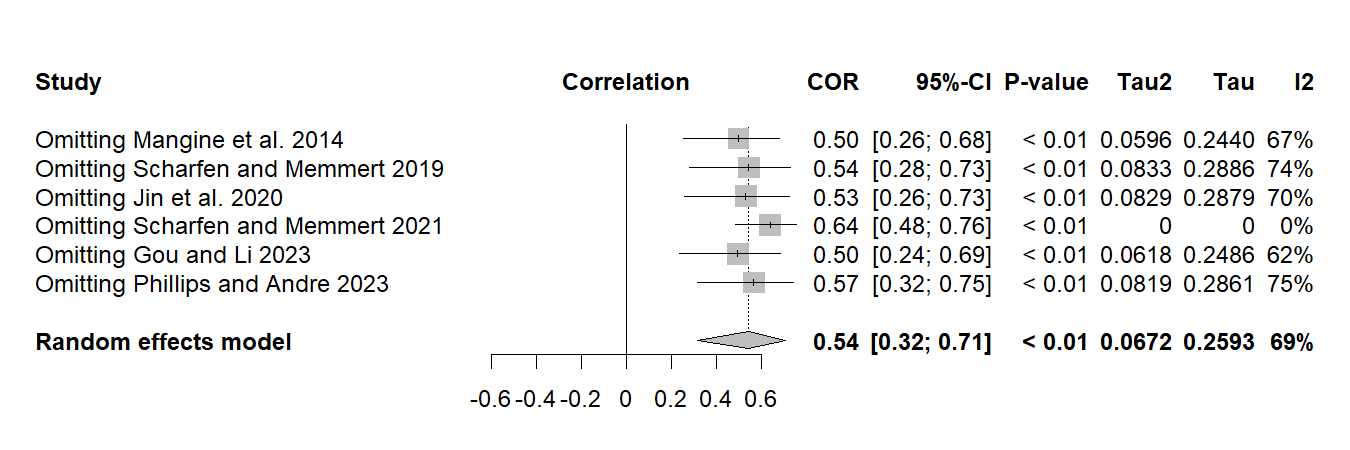
**

Figure 5.7 Sensitivity analysis (leave-one-out) of the pooled correlation between **Multiple Object Tracking** and sport-specific performance using a random-effects model

**
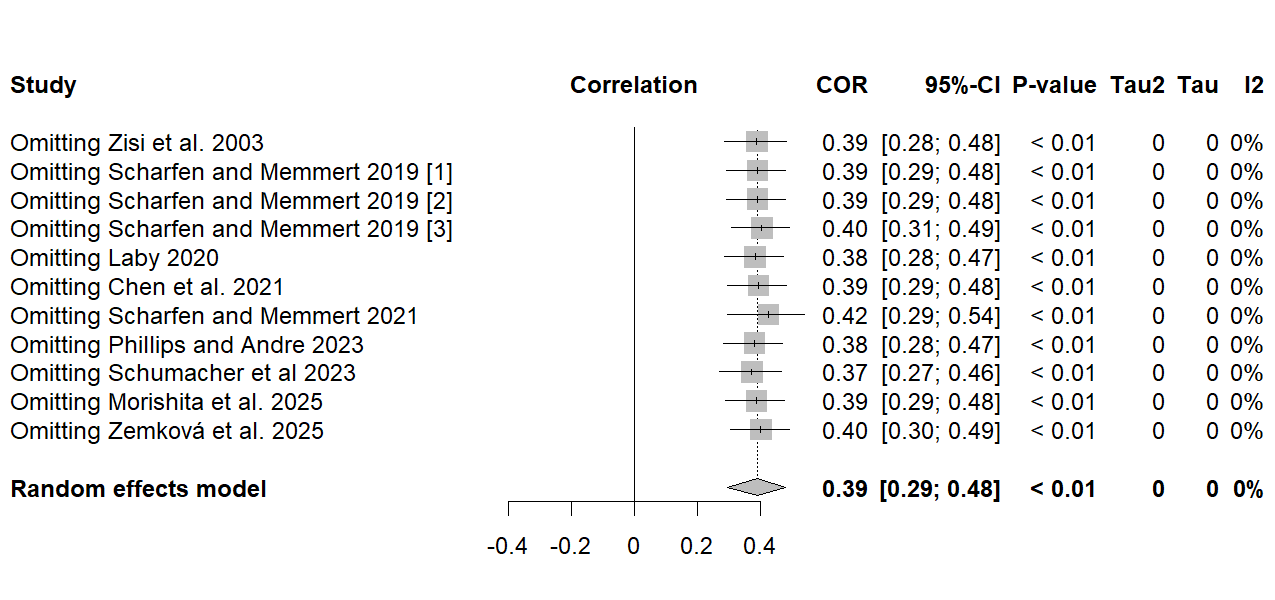
**

Figure 5.8 Sensitivity analysis (leave-one-out) of the pooled correlation between **Visual Attention** and sport-specific performance using a random-effects model


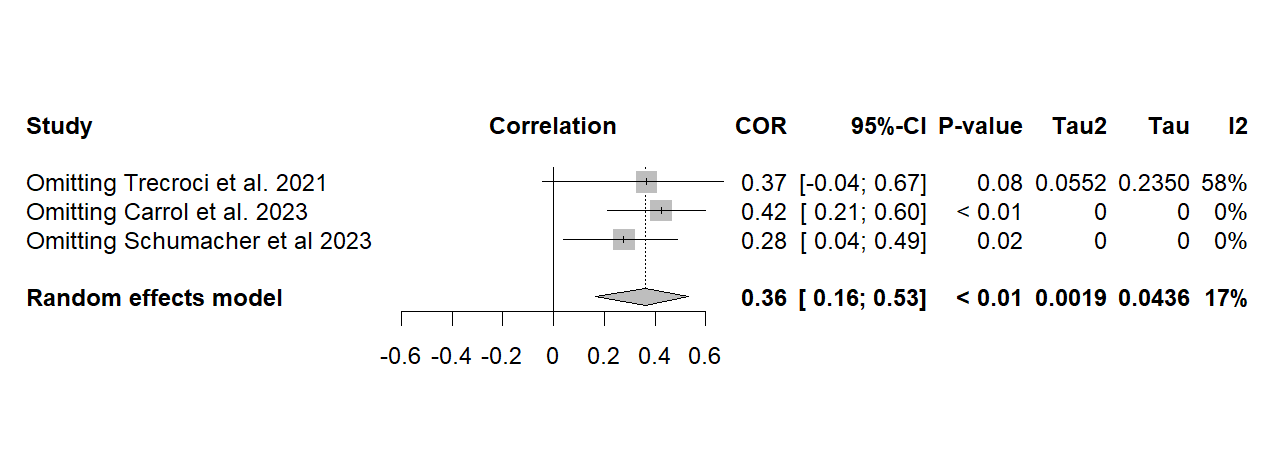


Figure 5.9 Sensitivity analysis (leave-one-out) of the pooled correlation between **Visual Search** and sport-specific performance using a random-effects model


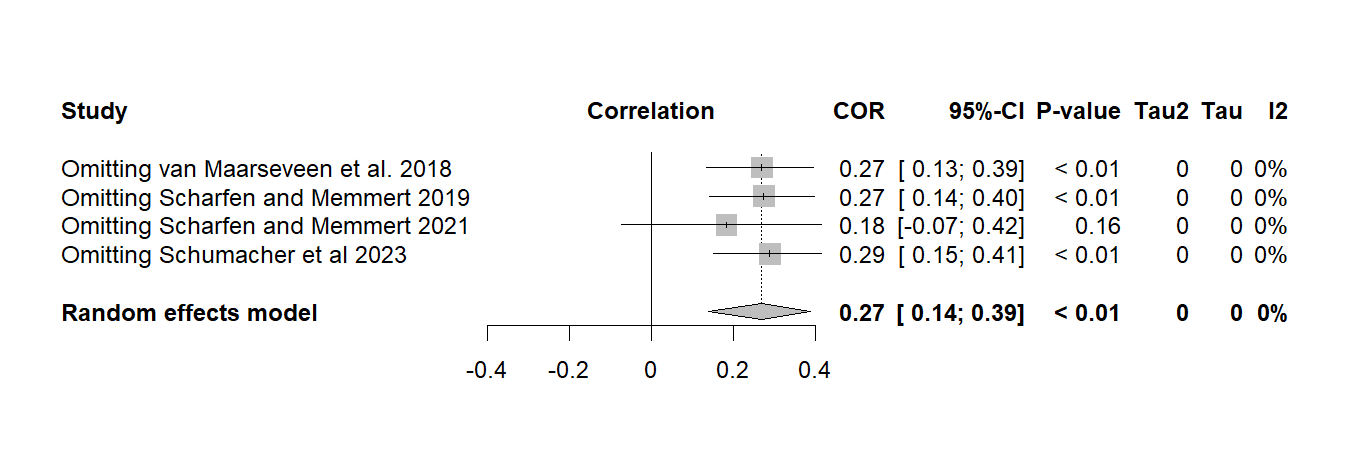


Figure 5.10 Sensitivity analysis (leave-one-out) of the pooled correlation between **Visual Working Memory** and sport-specific performance using a random-effects model
